# Efficacy of dental stem cell–derived exosomes for pulp regeneration: a systematic review of clinical, animal, and in vitro studies

**DOI:** 10.1007/s11033-026-11547-x

**Published:** 2026-02-24

**Authors:** Julia Godoi-Lopes, Victor Hugo Alves Ribeiro-Silva, Larissa Gregório Candido do Prado, Igor Bassi Ferreira Petean, Lais Valencise Magri, Fabiane Carneiro Lopes-Olhê, Carla Renata Sipert, Jardel Francisco Mazzi-Chaves

**Affiliations:** 1 Department of Restorative Dentistry, School of Dentistry of Ribeirão Preto, University of São Paulo, São Paulo, Brazil; 2 Department of Restorative Dentistry, School of Dentistry, University of São Paulo, São Paulo, Brazil; 3 Department of Endodontics Ribeirão Preto School of Dentistry, University of São Paulo (USP), Av. do Café, s/n, Ribeirão Preto, São Paulo, 14020-904 Brazil

**Keywords:** Exosomes, Regenerative endodontics, Dental pulp stem cells, Pulp–Dentin complex

## Abstract

Exosomes derived from dental stem cells (DSC-Exos) are nanovesicles (~ 30–150 nm) that mediate angiogenesis, odontoblastic differentiation, and immune regulation, positioning them as promising cell-free candidates for pulp–dentin regeneration. This PROSPERO-registered systematic review evaluated the regenerative potential of DSC-Exos and the methodological factors influencing outcomes. A comprehensive search of PubMed, Scopus, Web of Science, and Embase was conducted through July 2025; risk of bias was assessed using RoB 2.0, SYRCLE, and QUIN tools. Thirteen studies fulfilled the inclusion criteria, most of which employed exosomes derived from human dental pulp stem cells, stem cells from human exfoliated deciduous teeth and stem cells from the apical papilla. DSC-Exos consistently promoted cell proliferation and migration while upregulating odontogenic (DSPP, DMP1) and angiogenic (VEGF, CD31) markers. Animal models demonstrated vascularized pulp-like tissue and organized tubular dentin formation, significantly enhanced by donor-cell preconditioning and biodegradable carriers. Mechanistic investigations linked these outcomes to noncoding RNAs and signaling axes like miR-26a/TGF-β and circ_0003057/EIF4A3/ANKH. A 24-month clinical pilot confirmed restored pulp sensitivity without safety concerns. Nevertheless, the overall certainty of evidence remains moderate to low, mainly due to substantial methodological heterogeneity, limited sample sizes and insufficient reporting of critical parameters such as exosome characterization, dosing strategies and operator calibration. Collectively, the findings indicate that DSC-Exos represent a high-potential biomimetic strategy for pulp–dentin regeneration; however, standardized characterization protocols in line with MISEV guidelines, dose harmonization and well-designed long-term controlled clinical trials are essential before cell-free endodontic therapies can be reliably incorporated into clinical practice.

**Supplementary Information:**

The online version contains supplementary material available at 10.1007/s11033-026-11547-x.

## Introduction

The inability to restore a functional pulp with adequate vascularization and innervation in immature or non-vital teeth compromises both biological integrity and clinical longevity, making regeneration of the pulp–dentin complex a priority in contemporary endodontics [1, 2]. Although procedures such as apexification and revitalization offer benefits, they frequently yield reparative mineralized tissue without reconstitution of a functional pulp niche, thereby limiting restoration of homeostasis and full organ function [3].

In this context, dental-derived mesenchymal stem cells, including dental pulp stem cells (DPSCs), stem cells from human exfoliated deciduous teeth (SHED) and stem cells from the apical papilla (SCAP) demonstrate odontogenic and vasculogenic potential in experimental models [4]. However, clinical translation of cell-based therapies faces substantial barriers, including standardization of cell supply, donor-dependent phenotypic variability, survival within inflammatory microenvironments, and regulatory requirements for manufacturing and quality control [5]. Moreover, recent evidence suggests that the regenerative actions of mesenchymal stem cells (MSCs) are largely mediated by their secretome, particularly extracellular vesicles (EVs), with emphasis on small extracellular vesicles [6].

Small extracellular vesicles (sEVs), which includes exosomes, are small nanoparticles (≈ 30–150 nm in diameter) originating from intracellular multivesicular bodies [7, 8]. These vesicles encapsulate bioactive molecules, including proteins, lipids, transcription factors, and nucleic acids that mediate intercellular communication and modulate recipient cell behavior [9, 10], thereby recapitulating many effects of their cells of origin. Accumulating evidence indicates that dental stem cell-derived exosomes modulate angiogenesis, odontoblastic differentiation, matrix remodeling, and immunoregulation, acting as independent biological agents, adjuncts to cell therapies, or components of controlled-release systems such as hydrogels and microspheres [9, 11, 12].

Preclinical studies report consistent upregulation of odontogenic and angiogenic markers, as well as formation of vascularized, pulp-like tissue and tubular dentin in animal models [9, 12]. However, a recent review by Ahmad et al. (2025) underscored marked heterogeneity arising from differences in cell source, isolation and characterization methods, dose units employed, and the histological and functional criteria used to evaluate outcomes [9]. These gaps highlight the need for an evidence-based synthesis. A systematic review could critically appraise and synthesize existing data, evaluate methodological quality and risk of bias, and propose harmonized experimental and regulatory criteria to guide future preclinical and clinical studies. As emphasized by Ahmad et al. (2025), by systematically addressing these constraints, the therapeutic potential of DSC-Exo-based regenerative endodontic therapies can be fully realized, facilitating comprehensive structural and functional restoration of the pulp–dentine complex [9].

Moreover, experimental variables such as hypoxic or inflammatory preconditioning and the choice of delivery carriers employed can markedly modulate exosome bioactivity [13–15]. Given this variability, a critical synthesis that integrates mechanistic evidence, assesses methodological quality, and establishes criteria for experimental and regulatory harmonization is essential, including adherence to MISEV (Minimal Information for Studies of Extracellular Vesicles) guidelines, standardization of dose units, and systematic use of appropriate controls [9, 16].

Accordingly, this systematic review sought to answer the following PICO question: in preclinical and clinical models (P), does administration of dental stem cell-derived exosomes (I), compared with controls or standard interventions (C), promote structural and functional pulp regeneration (O)? The outcomes observed include evaluation of vascularized pulp-like tissue formation, the presence of an organized odontoblastic layer, dentinal repair, expression of relevant molecular markers, and clinical or radiographic indicators. In addition to synthesizing efficacy and mechanisms, this review examines risk of bias and discusses operational recommendations to guide experimental standardization, validation of adequately powered studies, and safe progression toward clinical application.

## Materials and methods

### Study design and registration

This systematic review was conducted and reported in accordance with the PRISMA 2020 statement [17]. The protocol was prospectively registered in PROSPERO (CRD420251083479) [18]. All key methodological decisions, including databases, eligibility criteria, search strategy, data extraction items, risk of bias assessment tools, and synthesis plan, were pre-specified in the protocol to minimize bias and ensure reproducibility.

### Information sources and search strategy

A comprehensive electronic search was performed in four bibliographic databases: PubMed (NCBI), Embase (Elsevier/Embase.com), Scopus (Elsevier) and Web of Science Core Collection (Clarivate Analytics) on 29 July 2025. The search covered all records available up to that date, with no publication-date restrictions; however, due to limitations in translation resources, inclusion was restricted to studies that could be reliably translated. Search strategies combined controlled vocabulary (MeSH/Emtree) and free-text terms tailored to each database’s syntax (Supplementary Fig. 1). Full search strategies (literal queries per database) and the number of records retrieved per database are presented in Supplementary Table S1. A total of 217 records were identified: PubMed (*n* = 138), Scopus (*n* = 40), Web of Science (*n* = 22), and Embase (*n* = 17). Database access was provided through the CAPES/CAFe consortium (CNPq, Brazil).

### Study selection

All records were imported into Rayyan software, which was used for automatic duplicate removal and screening. After excluding 72 duplicates (71 across databases and 1 within a database), 145 unique records remained. During title and abstract screening, 118 articles were excluded for predefined reasons: systematic reviews (*n* = 50), inadequate scope (*n* = 54), studies not involving exosomes derived from dental stem cells (*n* = 10), and other reasons such as conference abstracts, case reports, or annual reviews (*n* = 4). A total of 27 full-text articles were selected for assessment; one was in Chinese and was therefore excluded and documented in the PRISMA flow diagram. Of the 26 articles assessed in full text, 13 were excluded due to ineligible study design, irrelevant interventions/outcomes, or insufficient data, resulting in 13 studies included.

Inter-rater agreement during title and abstract screening was Cohen’s κ = 0.82, indicating substantial agreement. For the pilot data extraction, agreement was Cohen’s κ = 0.80. Discrepancies at any stage were resolved by consensus between the two reviewers or, when necessary, by a third reviewer.

**Fig. 1:**
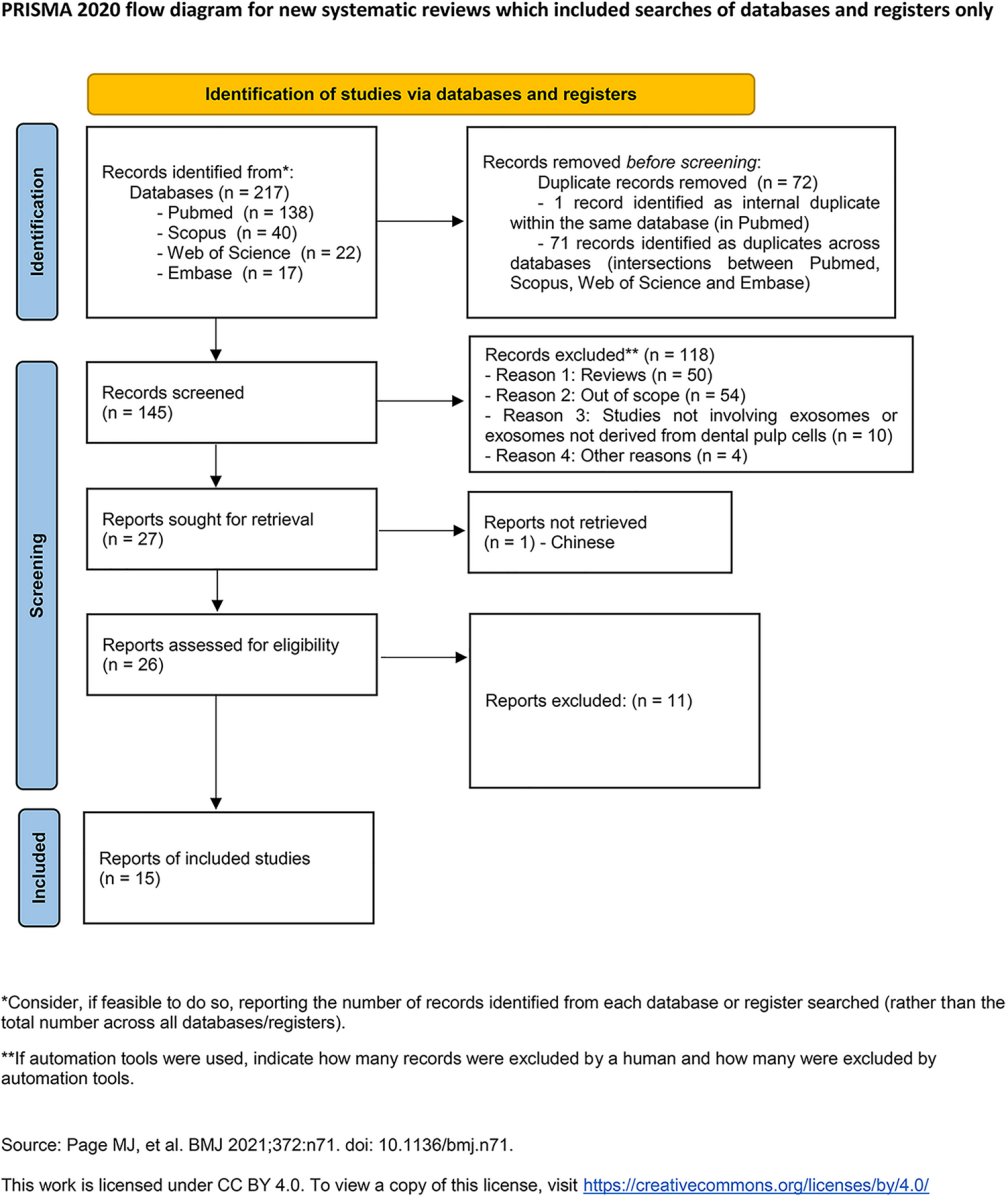
PRISMA Flow Diagram of Study Selection

Deduplication was performed in Rayyan using automatic matching by DOI and title/author metadata, followed by manual verification to remove duplicates not identified automatically. All counts for each stage are presented in the PRISMA flow diagram (Fig. 1).

### Eligibility criteria

The definition of the eligibility criteria followed the principle of maximizing sensitivity in identifying relevant evidence on the regenerative effects of dental stem cell–derived exosomes in models of pulpal injury or necrosis in permanent teeth, while simultaneously preserving sufficient specificity to exclude studies that do not allow the observed effects to be causally attributed to exosomes or that employ experimental models, interventions, or outcomes outside those defined in the PICO question (Table 1).

**Table 1:** Eligibility criteria according to the PICO framework

Domain	Criteria
Population/Model	Studies employing in vitro (cellular) and in vivo (animal) models, as well as clinical investigations, that examine pulp regeneration using dental-origin cells or tissues. Accepted cellular sources include dental pulp stem cells (DPSC), stem cells from human exfoliated deciduous teeth (SHED), stem cells from the apical papilla (SCAP), and periodontal ligament stem cells (PDLSC).
Intervention	Isolated and characterized exosomes derived from dental stem cells, administered either alone or in combination with vehicles or scaffolds. To be eligible, studies were required to report the method of exosome isolation (e.g., differential ultracentrifugation, size-exclusion chromatography, precipitation kits) and to present at least two complementary characterization techniques (for example, transmission electron microscopy (TEM), nanoparticle tracking analysis or dynamic light scattering (NTA/DLS), and protein marker detection such as CD63, CD9, or TSG101 by Western blot (WB) or flow cytometry).
Comparison	Comparators included no-exosome controls (vehicle), alternative interventions, or standard care. Studies lacking a formal comparator (descriptive experimental reports) were considered when they provided relevant primary data for the review question.
Outcomes	Primary outcome: formation of pulp-like tissue assessed by histological or histochemical analyses. Secondary outcomes: angiogenesis/neovascularization, odontoblastic differentiation (e.g., expression of DSPP, DMP1, ALP), radiographic or micro-CT indicators of tissue regeneration, functional markers, and adverse events reported in clinical studies. Follow-up durations were extracted and reported in standardized units (weeks or days).
Study Design	Clinical studies, in vivo studies, and three-dimensional in vitro models.

Inclusion criteria: studies investigating the application of exosomes (small extracellular vesicles, ≈ 30–150 nm) in models of pulpal injury or necrosis of permanent teeth were included, encompassing clinical studies in humans (randomized and non-randomized), controlled in vivo animal studies, and three-dimensional in vitro models reproducing relevant structural or functional aspects of the dental pulp. Only studies that clearly and reproducibly described the exosome isolation method and reported at least two objective vesicle characterization techniques (such as trans­mission electron microscopy, nanoparticle tracking analy­sis, dynamic light scattering, or identification of specific markers by Western blot or flow cytometry) were consid­ered eligible. Studies in which exosomes were administered alone, combined with biomaterials (hydrogels, fibrin, porous scaffolds, or microspheres), or co-administered with cells were included, provided that the experimental design allowed the contribution of exosomes to be isolated or meaningfully interpreted. Comparator groups included placebo or vehicle, no treatment, conventional regenerative endodontic procedures (revascularization/revitalization), or alternative experimental interventions; uncontrolled studies were included only when they reported quantitative histological or molecular outcomes that allowed inference of a regenerative effect. Eligible outcomes comprised histological evidence of pulp-like tissue formation (vascularized connective tissue, odontoblast-like layer, or tubular dentin deposition), expression of odontogenic or angiogenic markers (such as DSPP, DMP-1, Runx2, VEGF, CD31), or relevant clinical and radiographic functional measures. With respect to language, publications in English and Portuguese were eligible; publications in other languages were considered only if a verified translation was available, and no initial time restriction was applied in order to capture the full body of evidence available up to the predefined search cutoff date.

Exclusion criteria: studies using conditioned medium without explicit isolation and characterization of extracellular vesicles were excluded, as the observed effects could not be specifically attributed to exosomes; studies employing exosomes derived from non-dental sources were also excluded, unless direct comparisons allowed discrimination of their effects. Two-dimensional in vitro models that did not reproduce three-dimensional or functionally relevant aspects of pulp tissue were excluded, as were publications that failed to clearly and reproducibly report exosome isolation and characterization methods. Studies focusing exclusively on generic cellular outcomes, such as viability or proliferation, without assessment of odontogenic or angiogenic markers or histological or radiographic evidence related to pulpal regeneration were excluded. In addition, book chapters, letters to the editor, expert opinions, conference abstracts without full-text availability, and systematic reviews were excluded from the synthesis; systematic reviews were consulted solely for reference tracking and did not contribute to the primary quantitative synthesis.

### Data extraction

The data extraction was carried out in a systematic and reproducible manner by two independent reviewers, in accordance with the prospectively registered protocol. A standardized data-extraction form was developed in an electronic spreadsheet (Microsoft Excel) and, prior to the final extraction, a calibration pilot was performed on approximately 10% of the potentially eligible publications, which were randomly selected. The pilot aimed to assess the clarity of the form fields, the applicability of the coding rules, and inter-rater agreement. The Cohen’s kappa coefficient obtained in the pilot was κ = 0.80, a value interpreted as substantial agreement, which permitted continuation of the extraction process.

During the pilot, terminological ambiguities and fields that were difficult to standardize were identified, particularly those relating to the description of the experimental model, the characterization of the exosome intervention, and heterogeneity in the reporting of evaluated outcomes. These limitations prompted operational adjustments to the form and to the extraction manual, including the standardization of closed categories whenever possible and the inclusion of a specific code for “Not reported,” together with instructions to record complementary textual descriptions whenever information was not presented in a structured format by the study authors. In the final spreadsheet, these textual entries were retained in full to ensure transparency and fidelity to the original data, and were subsequently organized for the purposes of narrative synthesis.

The extracted variables comprehensively encompassed bibliographic information, methodological characteristics, a detailed description of the exosome intervention, and the main outcomes related to pulp regeneration. The following data were collected: publication identification (article title, authors, and year), study design, experimental model (clinical, in vivo, or three-dimensional in vitro), source of exosomes (DPSCs, SHED, SCAP, PDLSCs, among others), type of vesicle reported, detailed description of the intervention (including mode of application and whether combined with biomaterials or other approaches), presence or absence of a control group and its description, outcomes assessed (histological, molecular, radiographic, or functional), principal results, and the authors’ conclusions. Whenever applicable, the existence of comparative groups and the analytical methods used to assess outcomes in the included studies were explicitly recorded.

Predefined rules were established to address inconsistencies and missing data: when information essential for the characterization of the study or the intervention was not available in the main text or in supplementary materials, the corresponding field was uniformly coded as “Not reported,” without inference, extrapolation, or imputation. Qualitative descriptions provided by the authors were preserved in full as textual data, respecting the methodological heterogeneity intrinsic to the experimental and translational studies included in this review.

Discrepancies between the two reviewers during data extraction were initially resolved by consensus through joint discussion and re-evaluation of the original study. If disagreement persisted, a third independent reviewer was consulted to make the final decision. As an additional quality-control measure, a random sample of approximately 10% of the extractions was reviewed by a senior assessor to identify potential coding inconsistencies or transcription errors. The final database was stored in a secure digital environment with access controls and backups, and was exported in a nonproprietary format (CSV), ensuring traceability between the extracted data, the study-characterization tables, and the synthesis of results presented in subsequent sections of the review.

### Outcome definitions and unit of analysis

The primary outcomes were defined as molecular markers of odontogenesis and angiogenesis (e.g., DSPP, DMP-1, Runx2, VEGF, CD31), histological evidence of pulp-like tissue formation (vascularized connective tissue, odontoblast layer, dentinogenesis), and, when available, functional measures (sensitivity testing, radiographic parameters). Secondary outcomes included mechanistic correlates (regulatory miRNAs, signaling pathways), quantification of vascularization, and safety/tolerability data.

Due to substantial heterogeneity among studies regarding experimental design, exosome preparation and characterization, delivery systems, and outcome measures, meta-analysis was not deemed feasible. Therefore, a structured narrative synthesis was conducted, stratified by model type (in vitro, in vivo, clinical), intervention characteristics (cell source, isolation method, delivery system/carrier), and outcome domain (molecular, histological, functional). The primary unit of analysis corresponded to the experimental unit appropriate to each study design: in clinical studies, the patient or treated tooth as reported; in in vivo studies, the experimental animal or treated tooth/segment; and in in vitro studies, the sample or culture well considered by the authors. Whenever studies reported outcomes stratified by subgroups, the respective denominators were recorded and used in the corresponding analyses.

### Risk-of-bias assessment

The methodological quality and internal validity of the 13 included studies were evaluated using a multi-tool approach tailored to the specific experimental designs of each study, as recommended by the PRISMA 2020 statement. For the preliminary clinical evidence provided by the pilot study of Guo et al. (2021), the Revised Cochrane Risk-of-Bias Tool for Randomized Trials (RoB 2) was employed, assessing five critical domains: the randomization process, deviations from intended interventions, missing outcome data, measurement of the outcome, and selection of the reported result [20]. The preclinical components involving animal models were appraised using SYRCLE’s risk of bias tool, which adapts the Cochrane framework for laboratory animal research across ten domains, including sequence generation, baseline characteristics, allocation concealment, random housing, blinding of caregivers and outcome assessors, and reporting bias. Furthermore, given that the majority of studies utilized hybrid designs with substantial laboratory phases, the Quality Assessment Tool for In Vitro Studies (QUIN) was applied to evaluate the technical rigor of dental research experiments. The QUIN tool specifically focuses on 12 criteria, such as clearly stated aims, sample size calculation, detailed methodology, standardization, and statistical analysis, providing a structured percentage-based score to categorize studies into low, medium, or high risk of bias. The assessment process was conducted independently by two reviewers, with disagreements resolved through consensus-oriented discussion to ensure a robust appraisal of the evidence.

### Data synthesis

Given the clinical, methodological, and reporting heterogeneity among the included studies, particularly with respect to experimental and clinical study designs, units of analysis, model types (three-dimensional in vitro, in vivo, clinical), exosome preparation and characterization, delivery systems/carriers, mode and dose/volume of application, and the diverse outcome measures (molecular, histological, functional, vascularization quantification, and mechanistic correlates), a quantitative meta-analysis was not performed. Accordingly, the results were synthesized using a structured narrative approach in line with the PRISMA 2020 recommendations for systematic reviews without meta-analysis.

The synthesis was organized to allow systematic comparison across studies, prioritizing the description and frequency of primary outcomes (molecular markers of odontogenesis and angiogenesis, histological evidence of pulp-like tissue formation, and functional measures) and secondary outcomes (mechanistic correlates, vascularization quantification, and safety/tolerability data), as well as the technical context in which these results were obtained (cellular source of exosomes, isolation method, delivery system/carrier, mode of application, and any association with biomaterials). Whenever studies reported clear denominators, simple proportions of events or findings per study were presented without statistical aggregation across studies. Where appropriate, findings were stratified by model type, intervention characteristics, and outcome domain.

### Strategy for comparison and qualitative analysis

The comparison between interventions was conducted qualitatively and descriptively, based on the distribution of reported outcomes in the individual studies. Data were grouped according to pre-defined experimental and methodological categories, including outcome type (molecular, histological, functional), intervention characteristics (cell source of exosomes, isolation method, delivery system/carrier, mode and dose/volume of application), and study model (in vitro, in vivo, clinical).

When available, direct comparisons between intervention parameters within the same study were recorded; however, no statistical comparative measures were calculated due to the lack of methodological uniformity and the predominance of observational, in vivo, in vitro, and case-report study designs.

### Ethical considerations

All data analyzed were obtained from previously published studies. No new human participants or animals were involved; therefore, approvals from human research or animal welfare ethics committees were not applicable.

## Results

### Overview of the evidence base

The search and selection process resulted in 13 included studies; all reported in vitro data, 12 incorporated complementary in vivo animal experiments, 1 focused on a complex 3D in vitro model using decellularized matrices to simulate the pulpal environment [19], and 1 provided clinical observations involving the bioengineering of 15 avulsed teeth in human patients [20]. The predominant cell sources for EV isolation were human DPSCs, followed by SHED and SCAP. Systematic heterogeneity was observed in vesicle isolation methods, with differential ultracentrifugation being the gold standard used in 12 of the 13 studies, while precipitation kits (ExoQuick-TC) were utilized only by Diomede et al. (2022) [19]. Characterization techniques followed MISEV guidelines using NTA, TEM, and Western Blotting for markers such as CD63, CD9, CD81, TSG101, and Alix. Delivery platforms were diverse, utilizing natural matrices like collagen tapes and decellularized dental pulp (DDP) alongside synthetic systems including GelMA, chitin-based hydrogels (HPCH/CW), and PLGA-PEG-PLGA microspheres.

In addition to the 13 studies included in the primary synthesis, we identified 13 supplementary studies that provided critical methodological and mechanistic insights but were excluded from the main dataset for utilizing animal-derived cells, non-dental sources, or focusing strictly on isolated processes like angiogenesis without demonstrating complete pulp–dentin complex regeneration.

Details of excluded but contextually relevant studies, along with their methodological and mechanistic contributions, are provided in Supplementary Table S2.

### Detailed characterization of included studies

A detailed analysis of the 13 included studies, as consolidated in the summary of evidence, provided comprehensive information regarding the use of extracellular vesicles (EVs) in the regeneration of the pulp–dentin complex (Table 2). China led the scientific production in this field with 10 studies [20–29], followed by the United States with 2 studies [30, 31] and Italy with 1 study [19]. Most research utilized combined experimental designs, integrating in vitro assays to elucidate molecular mechanisms and in vivo models-primarily involving subcutaneous transplantation in nude mice or orthotopic models in rats-to validate functional tissue formation.

**Table 2:** Detailed characterization of included primary studies

Study Design	Country	Study Design	Exosome Source (Donor)	Pre- conditioning/Donor State	Recipient Cell Type	Isolation Method	Molecular Cargo/Mechanism	Vesicle Dose Metric & Regimen	Delivery System/Carrier	Mode of Application	Control Group	Animal Model/Site	Outcomes (Cat.)	Time	Key Findings	Notes
Huang et al., 2016 [30]	USA/University of Illinois	*In vitro e In vivo*	Human Dental Pulp Stem Cells (hDPSCs)	Growth vs. Odontogenic media	hDPSCs and Human Bone Marrow Mesenchymal Stem Cells (hBMMSCs)	Ultracentrifugation	p38 Mitogen-Activated Protein Kinase (MAPK) signaling	5–10 *µ*g/mL	Clinical-grade Collagen Tape	Scaffold- impregnated graft	Vehicle (Phosphate Buffered Saline)	Nude mice (Subcutaneous)	Molecular/Histological	2 weeks	Odontogenic-induced exosomes are superior inducers of pulp-like tissue; identification of caveolar uptake.	Mechanism involves Heparan Sulfate Proteoglycans (HSPGs).
Zhuang et al., 2020 [34]	China/China Medical University	*In vitro e In vivo*	Stem cells from Apical Papilla (SCAPs)	Basal state	Rat Bone Marrow Mesenchymal Stem Cells (BMMSCs)	Ultracentrifugation	Dentin Sialophosphoprotein (DSPP) induction	50 *µ*g/mL	Gelatin Sponge	Scaffold- impregnated graft	BMMSCs only (no exosomes)	Nude mice (Subcutaneous)	Molecular/Histological	12 weeks	SCAP-derived exosomes promoted specific dentinogenesis and vascularity in root fragments.	Highlighted specificity for DSPP over Alkaline Phosphatase (ALP).
Swanson et al., 2020 [31]	USA/University of Michigan	*In vitro e In vivo*	hDPSCs and MDPC-23 cell line	Odontogenic induction	hDPSCs and Mouse DPSCs (mDPSCs)	Differential Centrifugation	MAPK/Extracellular Signal-Regulated Kinase (Erk1/2)	600 *µ*g per 1 mg of Microspheres	PLGA-PEG-PLGA Microspheres	Intracanal (Paste application)	Glass Ionomer Cement/Blank Microspheres	Rat (Molar Pulpotomy)	Molecular/Histological	6 weeks	Controlled release induced a tubular reactionary dentin bridge; cross-species efficacy confirmed.	Use of D-Trehalose as a stabilizer to prevent vesicle aggregation.
Chen et al., 2021 [22]	China/Guangxi Medical University	*In vitro e In vivo*	Human Dental Pulp Stem Cells (hDPSCs)	Lipopolysaccharide (LPS) (1 *µ*g/mL)	Rat Bone Marrow Mesenchymal Stem Cells (BMSCs)	Ultracentrifugation	Transforming Growth Factor-beta (TGF-*β*) signaling	200 *µ*g/mL (in vivo dose)	Peptide Hydrogel (PuraMatrix)	Intracanal injection	Normal sEVs/Phosphate Buffered Saline	Rat (Pulpless root canal)	Molecular/Histological	30 days	LPS-preconditioned sEVs facilitated functional pulp healing rather than scar healing.	LPS preconditioning significantly increased vesicle protein yield.
Guo et al., 2021 [20]	China/Fourth Military Medical University	Preclinical e Clinical Pilot	Stem cells from Human Exfoliated Deciduous teeth (SHEDs)	Cell Aggregates	Human Dental Pulp Stem Cells (hDPSCs)	Ultracentrifugation	Odontogenic Microenvironmental Cues	2 × 10⁷ cells (as aggregate)	Decellularized Tooth Matrix (DTM)	Scaffold- impregnated graft	Traditional delayed replantation	Minipig & Human (Avulsed teeth)	Histological/Functional/Radiographic	12–24 months	Functional regeneration with restored sensation, vascularity, and continued root growth.	Note: Study refers to cells as “hDPSCs” but specifies isolation from deciduous teeth (SHEDs).
Wu et al., 2021 [23]	China/Fourth Military Medical University	*In vitro e In vivo*	Stem cells from Human Exfoliated Deciduous teeth (SHEDs)	Cell Aggregates	SHEDs and Human Umbilical Vein Endothelial Cells (HUVECs)	Ultracentrifugation	miR-26a/SMAD2/3 signaling	50 *µ*g/mL	Human tooth fragments	Scaffold- impregnated graft	GW4869 (Exosome inhibitor)	Nude mice (Subcutaneous)	Molecular/Histological	12 weeks	SHED-aggregate exosomes promote pulp angiogenesis via exosomal shuttling of miR-26a.	Double-staining (CD31/Mitochondria) confirmed host and donor cell contribution.
Diomede et al., 2022 [19]	Italy/University of Chieti- Pescara	In vitro	Human Dental Pulp Stem Cells (hDPSCs)	5-Azacytidine (5 *µ*M)	hDPSCs	Commercial Kit (ExoQuick-TC)	DNA Demethylation of odontogenic genes	24 *µ*g total protein	Decellularized Dental Pulp (DDP)	Scaffold- impregnated graft	Basal Medium (Control)	None (In vitro study)	Molecular/Histological	14 days	DDP combined with EVs and 5-Aza significantly enhanced odontoblastic phenotype markers.	Epigenetic modulation through inhibition of DNA methyltransferases.
Li et al., 2022 [25]	China/Fourth Military Medical University	*In vitro e In vivo*	Stem cells from Human Exfoliated Deciduous teeth (SHEDs)	Apoptosis (Staurosporine induced)	Endothelial Cells (ECs)	Gradient Centrifugation	TUFM protein/TFEB-autophagy pathway	20 mg/mL (in vitro)	Phosphate Buffered Saline Gel	Intracanal injection	Conventional Revascularization	Beagle Dogs (Incisors)	Histological/Functional/Radiographic	3 months	Apoptotic vesicles (apoVs) recruited host ECs via autophagy to induce revascularization.	Note: Labeled as hDPSCs by authors but isolated from deciduous pulp (categorized as SHEDs).
Li; Ge, 2022 [24]	China/Tongji University	*In vitro e In vivo*	Dental Pulp Stem Cells (DPSCs)	Pulpitis model (Inflammatory)	Mesenchymal Stem Cells (MSCs)	Sucrose cushion/Ultracentrifugation	lncRNA-Ankrd26/miR-150/TLR4 axis	Not reported	Conditioned Medium	Intrapulpal injection	Vehicle (Phosphate Buffered Saline)	Rat (Incisor injury)	Molecular/Histological	30 days	Exosomal lncRNA-Ankrd26 regulates the miR-150-TLR4 axis to promote pulp restoration.	Identified specific lncRNA acting as a “sponge” for microRNAs.
Wang et al., 2023 [28]	China/Wuhan University	*In vitro e In vivo*	Human Dental Pulp Stem Cells (hDPSCs)	Basal state	hDPSCs and Human Umbilical Vein Endothelial Cells (HUVECs)	Ultracentrifugation	p38 MAPK Signaling	80 *µ*g/mL	HPCH/CW Thermosensitive Hydrogel	Intracanal injection	hDPSCs only (no exosomes)	Nude mice (Subcutaneous)	Molecular/Histological	8 weeks	Chitin whisker reinforcement improved hydrogel stiffness (1.06 kPa), favoring pulp-like tissue.	CW reinforcement provides structural mimicry of the extracellular matrix.
Lu et al., 2024 [26]	China/Sun Yat-sen University	*In vitro e In vivo*	Stem cells from Human Exfoliated Deciduous teeth (SHEDs)	Odontogenic induction	Human Dental Pulp Stem Cells (hDPSCs)	Ultracentrifugation	AMPK/mTOR pathway	20 *µ*g/mL (in vitro)	Gelatin Methacryloyl (GelMA) Hydrogel	Intracanal injection	Basal Exosomes/Phosphate Buffered Saline	Nude mice (Subcutaneous)	Molecular/Histological	8 weeks	Odontogenic-induced SHED exosomes combined with GelMA facilitated dense mineralized tissue.	High-throughput sequencing identified 51 differentially expressed miRNAs in OM-EVs.
Wang et al., 2025 [27]	China/Guangzhou Medical University	*In vitro e In vivo*	Human Dental Pulp Stem Cells (hDPSCs)	Odontogenic induction	hDPSCs	Ultracentrifugation	circ_0003057/EIF4A3/ANKH axis	100 *µ*g per fragment	Human dental fragments	Subcutaneous transplant	NC/hDPSCs only (no exosomes)	Nude mice (Subcutaneous)	Molecular/Histological	8 weeks	Exosomal circ_0003057 binds EIF4A3 to upregulate the parental gene ANKH, inducing differentiation.	Identified ANKH (Ankyrin motif) as a key regulator of odontoblast differentiation.
Wang et al., 2025 [29]	China/Huazhong University	*In vitro e In vivo*	Human Dental Pulp Stem Cells (hDPSCs)	Early Odontogenesis (3 days)	hDPSCs and Human Umbilical Vein Endothelial Cells (HUVECs)	Differential Centrifugation	Developmental cues (Odontogenic)	50 *µ*g/mL	Matrigel	Subcutaneous transplant	Normal DPSC-Exos	Nude mice (Subcutaneous)	Molecular/Histological/Functional	8 weeks	DPSC-Od-Exos promoted complete pulp-dentin regeneration, including neurogenesis (NF200).	First study to emphasize exosome-mediated neurogenic recovery in pulp regeneration.

Regarding the methodological parameters, differential ultracentrifugation was the gold-standard isolation method used in 12 of the 13 studies to ensure high purity of the exosomal fraction. The study by Diomede et al. (2022) was the sole exception, employing a commercial precipitation kit (ExoQuick-TC) [19]. Physical characterization across all evidence confirmed spherical or “cup-shaped” nanovesicles with diameters ranging from 30 to 200 nm, validated by Transmission Electron Microscopy (TEM) and Nanoparticle Tracking Analysis (NTA). Protein identity was confirmed by the enrichment of classical markers such as CD9, CD63, CD81, Alix, and TSG101, while the absence of Calnexin ruled out cellular contamination.

The cellular sources were strictly human-derived, including human Dental Pulp Stem Cells (hDPSCs), Stem cells from Human Exfoliated Deciduous teeth (SHEDs), and Stem Cells from Apical Papilla (SCAPs). A significant finding was the necessity of cellular preconditioning to enhance the therapeutic potency of the cargo. Successful protocols included odontogenic induction [26, 27, 29] [30], cell aggregate culture [20, 23], LPS-induced inflammation [22], epigenetic modulation [19], and Staurosporine-induced apoptosis to generate apoptotic vesicles (apoVs) [25].

In terms of delivery systems (scaffolds), the studies demonstrated a transition to injectable and bioactive hydrogels. Materials included 5% GelMA [26], HPCH/CW hydrogels reinforced with chitin whiskers [28], and PuraMatrix Peptide Hydrogel [22]. Advanced engineering was represented by PLGA-PEG-PLGA microspheres with a “sphere-in-sphere” morphology, stabilized by 2 mM D-trehalose to protect the exosomal RNA cargo for up to 12 weeks of sustained release [31]. Additionally, decellularized matrices-such as Decellularized Dental Pulp (DDP) and Treated Dentin Matrix (TDM)-were used to reconstruct the native developmental niche.

The assessed outcomes highlighted odontogenic and angiogenic markers such as DSPP, DMP1, ALP, CD31, and Runx2. Histological evidence consistently showed the formation of pulp-like vascularized connective tissue, an organized odontoblast-like layer, and the deposition of tubular dentin. Mechanistic evaluations identified specific regulatory axes, including p38 MAPK [28, 30], AMPK/mTOR [26], circ_0003057/EIF4A3/ANKH [27], lncRNA-Ankrd26/miR-150/TLR4 [24], and miR-26a/SMAD2/3 [23]. Finally, high-level evidence from a clinical pilot study [20] confirmed systemic safety and functional recovery of pulp sensitivity in human patients over 24 months.

### DPSCs-derived exosomes

Studies utilizing human Dental Pulp Stem Cells (hDPSCs) constitute the empirical core of the selected evidence, demonstrating a methodological progression from initial signaling elucidation to advanced delivery engineering and orthotopic validation (Supplementary Table S3).

Huang et al. (2016) established a foundational framework by isolating exosomes from hDPSCs cultured in either growth medium (DPSC-Exo) or odontogenic induction medium (DPSC-OD-Exo) for 4 weeks. These vesicles, approximately 100 nm in diameter and CD63/CD9 positive, were found to bind specifically to matrix proteins such as type I collagen and fibronectin through a controlled, saturable mechanism. The study demonstrated that hDPSCs endocytose these exosomes via a caveolar/lipid raft-mediated pathway-inhibited by heparin and MBCD-which subsequently triggers the p38 MAPK signaling cascade and nuclear translocation of pP38. In vivo results from a tooth root slice model in nude mice confirmed that DPSC-OD-Exos are superior inducers, promoting the regeneration of vascularized pulp-like tissue and the deposition of DMP1 and DPP at the soft tissue-dentin interface [30].

Swanson et al. (2020) significantly advanced delivery technology by encapsulating hDPSC-derived exosomes into biodegradable PLGA-PEG-PLGA triblock copolymer microspheres (EXO-MS). Utilizing a modified double emulsion method and 2 mM D-trehalose as a cryoprotectant/stabilizer, they achieved a unique “sphere-in-sphere” morphology that protected the exosomal RNA cargo for a sustained release period of up to 12 weeks. In a rat pulpotomy model, this system mediated the formation of a dense, organized dentin bridge with authentic dentinal tubules and a rich collagenous matrix, effectively protecting the underlying pulp tissue [31].

Chen et al. (2021) explored the impact of the inflammatory microenvironment by isolating small extracellular vesicles (sEVs) from LPS-preconditioned hDPSCs (L-sEVs). Preconditioning with 1 µg/mL LPS significantly increased the protein yield of the vesicles and enriched their capacity to activate TGF-β signaling. In an orthotopic rat model of a pulpless root canal, the delivery of L-sEVs via a PuraMatrix peptide hydrogel facilitated functional pulp healing, characterized by the formation of vascularized connective tissue and the expression of DSPP, rather than the disorganized scar-like tissue observed in controls [22].

Diomede et al. (2022) investigated the synergy between paracrine signaling and epigenetic modulation using a system involving decellularized dental pulp (DDP) and 5-Azacytidine (5-Aza). They demonstrated that hDPSC-derived EVs, when combined with 5-Aza (5 µM), induce the DNA demethylation of odontogenic genes, significantly upregulating the expression of ALP, RUNX2, COL1A1, DMP1, and DSPP. Confocal and SEM analyses confirmed that this combined treatment facilitates the robust recellularization of the DDP matrix, creating a highly biomimetic and growth-permissive environment for endodontic regeneration [19].

Li & Ge (2022) focused on the molecular determinants of pulp restoration, identifying the exosomal long non-coding RNA lncRNA-Ankrd26 as a vital mediator. Their research revealed that lncRNA-Ankrd26 acts as a molecular sponge for miR-150, thereby derepressing TLR4 expression and promoting the migration and odontogenic differentiation of recipient mesenchymal stem cells. In a rat incisor injury model, the intrapulpal injection of these exosomes significantly enhanced tissue restoration, providing causal evidence that specific exosomal non-coding RNAs coordinate cell-cell interactions during repair [24].

Wang et al. (2023) emphasized the physical requirements of the regenerative niche by developing a thermosensitive chitin-based hydrogel (HPCH) reinforced with chitin whiskers (CW). The CW reinforcement optimized the hydrogel’s mechanical stiffness to 1.06 kPa, closely mimicking the native dental pulp. This system ensured the sustained release of hDPSC exosomes, which activated the p38 MAPK pathway and facilitated the formation of vascularized pulp-like tissue within a root model, demonstrating the importance of structural mimicry in cell-free therapy [28].

Wang et al. (2025) elucidated a novel circRNA-mediated regulatory axis, showing that exosomal circ_0003057 is highly upregulated during the middle stages of odontogenic differentiation (day 7). Mechanistically, circ_0003057 binds to the RNA-binding protein EIF4A3 to facilitate its own export from the nucleus to the cytoplasm, where it upregulates its parental gene ANKH to promote mineralization. In vivo transplantation of human dental fragments loaded with these exosomes in nude mice resulted in significantly thicker and denser dentin-like tissue formation compared to basal exosome groups [27].

Wang et al. (2025) provided a comprehensive comparison between normal DPSC-Exos and those derived from cells undergoing early odontogenesis (DPSC-Od-Exos). They found that DPSC-Od-Exos (isolated after 3 days of induction) significantly outperformed normal exosomes in promoting neurogenesis, as evidenced by the sprouting of NF200-positive sensory nerve fibers and the expression of Nestin and GDNF. Their orthotopic fragments in nude mice showed a complete pulp-dentin complex featuring an organized odontoblast-like layer with cytoplasmic processes extending into newly formed tubular dentin and a high density of neurovascular structures [29].

Complementary studies that did not meet primary inclusion criteria (due to animal cell sources or isolated focuses) nonetheless provide vital mechanistic and methodological data.

Merckx et al. (2020) performed a comparative proteomic and functional analysis between DPSC and BM-MSC secretomes, finding that DPSCs are generally less potent in inducing endothelial cell chemotaxis and in ovo neovascularization compared to bone marrow sources. Their results highlighted that under certain conditions, EV-depleted conditioned medium might outperform isolated EVs, emphasizing the role of soluble factors in the total secretome [32].

Ivica et al. (2020) utilized the total population of human dental pulp cells (DPCs) to demonstrate that pulp-derived exosomes, when delivered in a diluted fibrin gel, significantly attract host MSCs and enhance their proliferation. Although excluded for using a heterogeneous DPC population rather than purified stem cells, the study validated fibrin as an effective injectable vehicle for clinical translation [33].

Zhang et al. (2020) focused on the temporal requirements of regeneration, demonstrating that hDPSC-derived EVs loaded into fibrin gels support rapid neovascularization (angiogenesis) within the root canal. By quantifying VEGF release and CD31 expression, they showed that EVs are more effective than conditioned medium (CM) in recruiting host endothelial cells during the early phases of repair [34].

Yan et al. (2022) explored the crosstalk between the pulp and periodontium, showing that EVs from inflammatory hDPSCs (iDPSC-EVs) regulate the differentiation of periodontal ligament stem cells (PDLSCs). They identified a significantly altered miRNA profile in inflammatory vesicles, specifically highlighting the miR-758-5p/LMBR1/BMP axis as a potential target for coordinating regional tissue repair [35].

Ganesh et al. (2023) proposed an “exosome-based cell homing” strategy using rabbit DPSCs, identifying an optimal functional dose of approximately 5 × 10⁸ particles/mL to promote migration and proliferation. Their small-RNA-seq profiling identified key pro-angiogenic miRNAs-such as miR-199a-3p and miR-21-5p- [36].

### SHED-derived exosomes

The evidence base utilizing Stem cells from Human Exfoliated Deciduous teeth (SHEDs) highlights their robust pro-angiogenic potential and developmental plasticity, often outperforming adult sources in terms of yield and specific miRNA enrichment (Supplementary Table S4).

Wu et al. (2021) demonstrated that SHED aggregates secrete specialized exosomes (SA-Exo) with a distinct miRNA signature compared to those from monocultures. Their investigation revealed that miR-26a is significantly enriched in SA-Exos and serves as a master regulator of vascularization. Mechanistically, these exosomes activate the TGF-β/SMAD2/3 signaling pathway in both recipient SHEDs and human umbilical vein endothelial cells (HUVECs), promoting endothelial differentiation and tube formation. In vivo, subcutaneous implantation of human root fragments in nude mice combined with SA-Exos resulted in a quantitative increase in CD31-positive vessels and the formation of an organized dentin-pulp complex, with double-staining confirming the contribution of both donor and host cells to the new vasculature [23].

Guo et al. (2021) provided the highest level of hierarchy in the evidence base by integrating SHED aggregates into a Decellularized Tooth Matrix (DTM) to revitalize avulsed teeth. This study established that the odontogenesis-related developmental microenvironment of deciduous pulp facilitates the delivery of high-density stem cells and self-produced extracellular matrix. Beyond robust in vitro characterization, this work included a clinical pilot study involving 15 avulsed teeth in human patients, demonstrating that the therapy not only restored thermal and electrical pulp sensitivity but also ensured systemic safety over a 24-month follow-up, with no adverse effects on liver or renal function [20].

Li et al. (2022) introduced a paradigm shift by utilizing apoptotic vesicles (apoVs) derived from SHEDs (which the authors initially labeled as hDPSCs but isolated from deciduous pulp). Induced via staurosporine treatment, these apoVs were found to carry the mitochondrial protein TUFM, which triggers the TFEB-mediated autophagy pathway in recipient endothelial cells. This mechanism accelerates revascularization, a critical prerequisite for sustained tissue formation. The study validated this approach in an orthotopic beagle dog incisor model, where intrapulpal injection of apoVs facilitated the recruitment of host endothelial cells and supported the regeneration of vital pulp-like tissue within three months [25].

Lu et al. (2024) focused on the optimization of “functional EVs” (OM-EV) by pre-differentiating SHEDs in odontogenic induction medium for 10 days. These OM-EVs displayed larger particle sizes and higher protein concentrations than basal vesicles, and were delivered using a 5% Gelatin Methacryloyl (GelMA) photocrosslinkable hydrogel to provide sustained release. High-throughput sequencing identified 51 significantly altered miRNAs, with eixos such as AMPK/mTOR being identified as central to the stimulation of recipient DPSCs. In vivo human dental fragments transplanted into nude mice showed that OM-EVs effectively upregulated markers like DSPP and DMP-1, resulting in the formation of dense mineralized tissue and an organized odontoblast-like layer [26].

Excluded studies provide additional layers of mechanistic data regarding SHED-derived vesicles, particularly in the realms of hypoxia response and immunomodulation.

Liu et al. (2022) explored the physiological microenvironment of root resorption by preconditioning SHEDs with hypoxia (2% O₂). They found that hypoxic exosomes (Hypo-exos) are larger and more concentrated than normoxic ones, carrying significantly higher levels of let-7f-5p and miR-210-3p. These miRNAs target AGO1 and EphrinA3, respectively, to enhance HUVEC proliferation and migration, facilitating neovascularization in ischemic scenarios. This study reinforces the strategy of using physiological stressors to enrich the exosomal cargo for targeted therapeutic effects [37].

Zuo et al. (2025) conducted a comparative analysis between SHED and adult DPSC sEVs, revealing that SHED vesicles have a 25.9 - fold higher expression of miR-200c-3p. This specific miRNA targets PTEN, thereby activating the PI3K/Akt signaling pathway to polarize macrophages toward a pro-healing M2 phenotype. Although excluded for focusing primarily on immunomodulation rather than tissue formation, this research establishes that SHED-derived exosomes are superior to DPSC-exosomes in creating a growth-permissive immune microenvironment essential for pulp regeneration [38].

### SCAP-derived exosomes

Stem cells from the apical papilla (SCAPs) are a unique population of mesenchymal stem cells residing in the tissue of immature permanent teeth, playing a critical role in continual root development. The evidence base demonstrates that SCAP-derived exosomes (SCAP-Exos) are particularly specialized in promoting directed dentinogenesis and managing the inflammatory microenvironment essential for the survival of the pulp–dentin complex (Supplementary Table S5).

Zhuang et al. (2020) provided definitive evidence of the dentinogenic specificity of SCAP-Exos. In this study, exosomes were isolated via differential ultracentrifugation (120,000 × g) and characterized as 120.6 nm spherical vesicles expressing CD9 and Alix. In vitro results demonstrated that SCAP-Exos (50 µg/mL) are endocytosed by bone marrow mesenchymal stem cells (BMMSCs), triggering a dose-responsive increase in Dentin Sialophosphoprotein (DSPP) expression and mineralized nodule formation without significantly altering classical osteogenic markers like ALP or Runx2. The in vivo validation utilized a subcutaneous transplantation model in nude mice, where SCAP-Exo-loaded gelatin sponges were inserted into human root fragments. After 12 weeks, histological analysis confirmed the formation of a new continuous dentine layer with an organized layer of polarized odontoblast-like cells and enhanced vascularization, effectively reconstructing the dentine–pulp interface [21].

The following studies provide critical mechanistic data regarding immunomodulation and the response to physiological stressors, though they were excluded from the primary synthesis for focusing on isolated processes rather than complete tissue regeneration.

Yu et al. (2022) explored the translational potential of SCAP-Exos in treating early pulpal inflammation. Utilizing an experimentally induced rat pulpitis model, the study demonstrated that intrapulpal injection of SCAP-Exos (1 µg/µL) significantly reduced inflammatory infiltration and promoted the accumulation of Foxp3 + regulatory T cells (Tregs). The molecular mechanism was identified as Tet2-mediated Foxp3 demethylation, which stabilized the Treg phenotype and increased IL-10 production. This work establishes SCAP-Exos as a potent cell-free tool for creating an immunologically permissive environment that facilitates subsequent regenerative processes in young permanent teeth [39].

Liu et al. (2023) investigated the enhancement of SCAP-Exo potency through hypoxic preconditioning (1% O₂). The research revealed that hypoxic exosomes are more abundant and significantly more effective than normoxic ones in promoting the proliferation, migration, and tube formation of human umbilical vein endothelial cells (HUVECs). The pro-angiogenic effect was found to be mediated by the HIF-1α/Notch1/JAG1/VEGF signaling axis, wherein exosomal JAG1 activates Notch signaling in recipient endothelial cells to upregulate VEGF production. These findings consolidate the strategy of using environmental stressors to “potentiate” the pro-vascularization capacity of SCAP-derived vesicles for endodontic engineering [13].

### Analytical characterization of vesicles: techniques, markers and observed limitations

The analytical characterization of extracellular vesicles (EVs) across the 13 included studies followed a rigorous but variable multi-analytical pipeline to ensure structural integrity and biological identity. While differential ultracentrifugation was the predominant isolation method used by the majority of authors [20–22, 26–30], alternative techniques were also employed. Li and Ge (2022) utilized a sucrose cushion combined with ultracentrifugation for higher purity [24], Li et al. (2022) applied gradient centrifugation for apoptotic vesicle (apoV) isolation [25], and Diomede et al. (2022) was the only group to utilize a commercial precipitation kit (ExoQuick-TC) [19].

Physical and morphological validation relied heavily on Transmission Electron Microscopy (TEM), which was explicitly reported in most included works to identify typical spherical or “cup-shaped” nanovesicles with distinct bilayer membranes) [21, 26, 29]. Quantitative size profiling was standardized via Nanoparticle Tracking Analysis (NTA), revealing mean diameters such as 71.22 nm [22], 120.6 nm [21], and 122–137 nm [26]. Notably, Lu et al. (2024) supplemented these findings with Atomic Force Microscopy (AFM) to confirm surface topography and a “saucer-like” morphology in a dry state [26]. For studies involving complex delivery systems, Scanning Electron Microscopy (SEM) was vital to visualize the interaction between vesicles and scaffolds, such as the “sphere-in-sphere” morphology of PLGA-PEG-PLGA microspheres [31].

Molecular identity was validated via Western Blotting (WB), focusing on tetraspanins (CD9, CD63, CD81) and biogenesis markers (TSG101, Alix, and Alix). To ensure the absence of cellular contamination, several studies verified that negative markers like Calnexin were not expressed in the EV fractions [26, 29]. Advanced cargo profiling, including high-throughput miRNA and circRNA sequencing, was selectively performed to identify specific regulatory eixos such as miR-26a [23], circ_0003057 [27] and lncRNA-Ankrd26 [24]. A significant methodological limitation observed across these 13 studies was metric heterogeneity, as dosage was inconsistently reported as total protein concentration (µg/mL), particle count (NTA), or cell-equivalent yield, complicating direct potency comparisons.

Supplementary insights from excluded studies provided additional technical depth regarding functional and enzymatic markers. Shi et al. (2023) introduced a functional potency metric by measuring the enzymatic activity of exosomal CD73/NT5E (20.67 mU/µg) [40]. High-resolution single-particle analysis via Nano-flow cytometry was utilized by Merckx et al. (2020) to confirm the presence of mesenchymal antigens (CD44, CD73, CD90) and the absence of hematopoietic markers [32]. Furthermore, studies like Zhang et al. (2020) and Ivica et al. (2020) utilized lipophilic dyes (PKH26, DiO) to demonstrate that vesicle internalization by recipient stem cells is a time-dependent process typically peaking between 6 and 12 h [33, 34]. Finally, research on physiological stressors highlighted that hypoxic preconditioning (1–2% O2) significantly increases total exosome yield and alters cargo enrichment with pro-angiogenic factors like let-7f-5p and miR-210-3p [13, 37].

### Delivery vehicles, Pharmacokinetic engineering and comparative effects

The evolution of cell-free regenerative endodontics has transitioned from the simple administration of extracellular vesicles (EVs) to the engineering of sophisticated delivery platforms designed to overcome the rapid clearance and low local accumulation inherent to “naked” exosomes. A recurrent finding among the 13 included studies is that successful in vivo outcomes depend as much on the delivery modality as on the exosome quality, with three primary approaches standing out: synthetic microspheres, injectable hydrogels, and decellularized organic matrices.

Swanson et al. (2020) pioneered advanced pharmacokinetic engineering by encapsulating odontogenic exosomes into biodegradable PLGA-PEG-PLGA triblock copolymer microspheres (EXO-MS) [31]. This system utilizes an amphiphilic polymer that creates hydrophilic reservoirs to protect the delicate exosomal cargo-specifically its RNA content-allowing for a controlled and sustained release period of 8 to 12 weeks. A critical innovation in this study was the use of 2 mM D-trehalose as a stabilizer, which acted as a molecular spacer to prevent vesicle aggregation and preserve bioactivity during the encapsulation process. Comparatively, while both diblock and triblock copolymers could encapsulate cargo, the triblock system demonstrated superior release kinetics, producing robust tubular dentin bridges and significantly higher mineralization in rat pulpotomy models compared to free exosome administration.

Injectable hydrogels represent the second major category of delivery vehicles, favored for their ability to fit complex root canal geometries and provide a mechanically permissive environment. Lu et al. (2024) utilized a 5% Gelatin Methacryloyl (GelMA) photocrosslinkable hydrogel to deliver odontogenic-induced SHED exosomes, demonstrating that this system prevents “explosive” burst release and maintains site retention, which effectively upregulates the odontogenic differentiation of recipient stem cells [26]. In a different approach to mechanical engineering, Wang et al. (2023) reinforced a thermosensitive hydroxypropyl chitin (HPCH) hydrogel with chitin whiskers (CW) [28]. This modification optimized the hydrogel’s stiffness to 1.06 kPa, closely mimicking the native dental pulp and facilitating the formation of vascularized pulp-like tissue with organized odontoblasts. Furthermore, Chen et al. (2021) demonstrated the efficacy of a PuraMatrix peptide hydrogel for the delivery of LPS-preconditioned vesicles, which facilitated functional healing and revascularization in a pulpless rat root canal model [22].

The third strategy involves the use of natural and decellularized matrices to simulate the developmental microenvironment. Huang et al. (2016) and Zhuang et al. (2020) utilized clinical-grade collagen tapes (Zimmer CollaCote) or gelatin sponges, observing that exosomes bind specifically to type I collagen and fibronectin in a saturable manner, which provides a stable substrate for the recruitment and differentiation of host cells [21, 30]. Scaling this approach to clinical levels, Guo et al. (2021) integrated cell aggregates into a Decellularized Tooth Matrix (DTM), illustrating that these organic scaffolds furnish powerful spatial cues that, when combined with exosomal secretions, restore pulp sensitivity and ensure long-term systemic safety in human patients [20]. Similarly, Diomede et al. (2022) utilized a decellularized dental pulp (DDP) system, demonstrating that the synergy between exosomal signaling and epigenetic modifiers (5-Azacytidine) induces robust recellularization and the expression of odontoblastic markers such as DSPP and DMP-1 [19].

Collectively, these studies indicate that sustained-release platforms (EXO-MS, GelMA, HPCH/CW) yield more consistent and organized repair results-characterized by authentic dentinal tubules and neurovascular sprouting - than the administration of free exosomes, which are prone to degradation in the proteolytic environment of an injured pulp.

Complementary studies that were excluded from the primary synthesis nonetheless provide critical corroboration for the translational importance of clinically viable carriers. Ivica et al. (2020) and Zhang et al. (2020) focused on the role of fibrin gels (diluted fibrin sealants), which are already FDA-approved for clinical use [33, 34]. Both studies demonstrated that the combination of fibrin and EVs significantly accelerates angiogenesis and collagen deposition in 3D co-culture models. Ivica et al. (2020) specifically highlighted that fibrinogen actively contributes to the observed effect by enhancing the migration and proliferation of host mesenchymal stem cells, suggesting that the carrier itself can act synergistically with the exosomal cargo [33]. Furthermore, Zhang et al. (2020) showed that fibrin-embedded EVs maintain a local therapeutic dose that induces the formation of hollow tubular structures within seven days, highlighting fibrin as a powerful, minimally invasive tool for “cell-homing” strategies in regenerative endodontics [34]. Other excluded works, such as Wang et al. (2025), utilized Matrigel for ectopic validation, further confirming that encapsulation is essential to reconstruct the complex neurovascular architecture of the pulp-dentin complex [29].

### Outcomes: odontogenesis, angiogenesis, dentinogenesis and neurovascular integration - detailed comparative synthesis

The regenerative outcomes across the 13 included studies demonstrate a coordinated biological response where extracellular vesicles (EVs) act as multifaceted signaling centers to orchestrate the reconstruction of the pulp-dentin complex. Huang et al. (2016) established that exosomes from odontogenic-induced human Dental Pulp Stem Cells (hDPSCs) are superior inducers of vascularized pulp-like tissue, specifically promoting the deposition of dentin matrix protein 1 (DMP1) and dentin phosphophoryn (DPP) at the soft tissue-dentin interface [30]. This directed action was further refined by Zhuang et al. (2020), who demonstrated that exosomes from stem cells from the apical papilla (SCAPs) selectively upregulate Dentin Sialophosphoprotein (DSPP) without significantly altering generalized osteogenic markers like ALP, resulting in the formation of a continuous new dentine layer lined with highly polarized, columnar odontoblast-like cells [21]. The structural integrity of these regenerative outcomes was confirmed by Swanson et al. (2020), whose sustained-release microsphere system mediated the formation of dense, organized dentin bridges characterized by authentic dentinal tubules and a collagen-rich extracellular matrix in rat pulpotomy models [31]. In inflammatory scenarios, Chen et al. (2021) proved that LPS-preconditioned small EVs facilitate functional pulp healing - evidenced by vascularized connective tissue and TGF-β signaling - rather than disorganized scar formation, effectively restoring a physiological structure within pulpless root canals [22].

The complexity of these outcomes scales significantly in higher-order models and clinical applications. Guo et al. (2021) reported the most comprehensive success to date, utilizing SHED aggregates in decellularized tooth matrices to achieve full-length pulp revitalization in humans, which was confirmed by the restoration of thermal and electrical sensitivity, CD31-positive vascularity, and continued root development over 24 months [20]. Parallelly, Wu et al. (2021) elucidated the angiogenic mechanism of SHED aggregates, showing that exosomal miR-26a activates the TGF-β/SMAD2/3 pathway to increase the density of CD31-positive vessels, with evidence that both donor and host cells contribute to the new microvasculature [23]. Epigenetic and physical niche modulations also play critical roles; Diomede et al. (2022) demonstrated that combining EVs with 5-Azacytidine induces DNA demethylation of odontogenic genes, facilitating the robust recellularization of decellularized pulp with cells exhibiting an odontoblastic phenotype [19]. Meanwhile, Li et al. (2022) introduced the role of apoptotic vesicles (apoVs), which trigger endothelial autophagy through the mitochondrial protein TUFM, leading to accelerated revascularization and significant radiographic dentin thickening in a beagle dog model [25].

Molecular precision in the restorative process is further evidenced by specific non-coding RNA pathways and mechanical optimization. Li & Ge (2022) identified the lncRNA-Ankrd26/miR-150/TLR4 axis as a vital driver for cell migration and pulp restoration in injury models [24], while Wang et al. (2023) showed that a chitin-based hydrogel with a native-like stiffness of 1.06 kPa is essential for exosomes to successfully trigger the formation of vascularized pulp-like tissue [28]. Emerging evidence from Lu et al. (2024) highlights the potential of “functionalized” SHED EVs to drive dense mineralized tissue formation through the AMPK/mTOR metabolic pathway, producing significant DSPP and DMP-1 expression [26]. Finally, the most recent advancements by Wang et al. (2025) and Wang et al. (2025) have expanded the scope to neurovascular integration; the former demonstrated that the exosomal circ_0003057/ANKH axis significantly increases the thickness and density of dentine-like tissue, while the latter provided the first definitive evidence of exosome-mediated neurogenesis, showing the sprouting of NF200-positive sensory nerve fibers within a complete pulp-dentin complex [27, 29].

Complementary studies provide additional depth to these findings by detailing early-stage kinetics and specific biological interactions. Zhang et al. (2020) observed that DPSC-derived EVs can induce the formation of hollow tubular vascular structures in as little as seven days, emphasizing the speed of the angiogenic response [34]. Ivica et al. (2020) and Chen et al. (2022) focused on the “cell homing” effect, demonstrating that pulp-derived vesicles are highly effective at recruiting host MSCs and SCAPs to the injury site, which is a prerequisite for any clinical tissue formation [33, 41]. The biochemical synergy of laser therapy was explored by Abdelgawad et al., 2022, who found that photobiomodulation significantly enhances exosome-mediated expression of MMP9 and TGF-β in dog models [42]. Furthermore, Zuo et al. (2025) and Yu et al. (2022) highlighted the immunomodulatory foundations of these outcomes, showing that the polarization of M2 macrophages and the stabilization of Foxp3 + Tregs create the necessary growth-permissive environment that allows odontogenesis and angiogenesis to proceed without the interference of chronic inflammation [38, 39]. Shi et al. (2023) provided a comparative benchmark by showing that exosomal adenosine signaling can produce dentin bridge outcomes comparable to Mineral Trioxide Aggregate (MTA), the clinical gold standard, but with potentially better biological integration [40]. Lastly, Yang et al. (2022) demonstrated that delivering the transcription factor NFIC via EVs can rescue impaired odontoblastic differentiation in periapical inflammatory environments, suggesting a path for treating complex cases like apical periodontitis [43].

### Preliminary clinical evidence and observed safety

Direct clinical evidence regarding the therapeutic application of extracellular vesicles (EVs) in endodontics is currently studied by Guo et al. (2021), which conducted a clinical pilot study involving 15 pediatric patients (ages 7–12) with avulsed permanent teeth [20]. This study utilized SHED aggregates integrated into a decellularized tooth matrix (DTM) to revitalize the pulpal space, providing a 24-month follow-up that established a significant benchmark for systemic safety and functional efficacy. Patients treated with this bioengineered construct demonstrated a complete restoration of thermal and electrical pulp sensitivity, alongside radiographic evidence of continued root development and increased root length. Crucially, the safety assessment performed at the 2-year mark included an exhaustive panel of blood chemistry and immune profiles, showing that parameters such as ALT, AST, total protein, urea, and creatinine remained within normal physiological ranges for all 15 patients. Furthermore, the percentages and total counts of T cells (CD4 + and CD8+), B cells, and NK cells were unaffected, confirming the absence of systemic toxicity or adverse immune reactions following the implantation of the exosome-secreting aggregates [20].

The safety profile of cell-free endodontic therapy is further supported by robust in vivo animal models among the included studies. Huang et al. (2016) emphasized that using odontogenic-induced exosomes is a safer alternative to the clinical administration of high-dose recombinant growth factors, such as BMP2, which has been associated with structurally abnormal bone formation and chronic inflammation. In their mouse root-slice model, exosome-mediated regeneration produced organized, vascularized pulp-like tissue without any reported ectopic mineralization or inflammatory infiltrate [30]. Similarly, Li et al. (2022) validated the safety of apoptotic vesicles (apoVs) in an orthotopic beagle dog model; after three months, the therapy facilitated the recruitment of host endothelial cells and supported Vitality without triggering adverse local responses in the periapical tissues [25]. The use of FDA-approved biodegradable polymers, such as the PLGA-PEG-PLGA triblock copolymers employed by Swanson et al. (2020), provides additional pharmacokinetic assurance, as these materials ensure a controlled release that limits systemic exposure and focuses the therapeutic effect within the root canal [31]. Mechanistic validation across these studies, including the use of the inhibitor GW4869, confirmed that the regenerative outcomes were specifically mediated by exosomal signaling, as blocking vesicle secretion abolished the therapeutic effect.

Excluded and complementary studies provide further theoretical and biochemical support for the safety of vesicle-based therapies compared to traditional cell transplantation. Merckx et al. (2020) and Ivica et al. (2020) noted that EVs present a higher safety profile than direct stem cell transplantation because they significantly mitigate the risks of tumorigenesis, immunorejection, and pathogen contamination inherent to living cell therapies [32, 33]. Furthermore, Abdelgawad et al., 2022 provided detailed biochemical monitoring in a canine model, observing that the combination of exosomes and photobiomodulation (PBM) did not adversely affect serum levels of ionized calcium or phosphorus, instead maintaining minerals at levels conducive to healthy reparative dentin formation [42]. These studies collectively reinforce the concept that cell-free vesicles act as stable, non-living signaling units that can be precisely dosed to achieve predictable healing without the complex regulatory and biological hurdles of cell-based “living” drugs.

### Comparative molecular signatures by exosome source

The molecular architecture of exosome-mediated regeneration is governed by source-specific signatures of non-coding RNAs and proteins that selectively activate pathways for tissue healing. Among the 13 included studies, human Dental Pulp Stem Cells (hDPSCs) provide exosomes that excel in orchestrating the final stages of mineralized matrix organization. A primary functional molecule identified is the circular RNA circ_0003057, which is highly enriched in odontogenic-induced hDPSC exosomes (DPSC-Od-Exos). This circular RNA acts by binding to the EIF4A3 protein, facilitating its own nuclear export to the cytoplasm, where it upregulates its parental gene ANKH, a critical regulator of odontoblast differentiation and phosphate transport necessary for mineralization. Complementing this genetic switch, hDPSC-derived vesicles utilize surface-bound CD73 (ecto-5’-nucleotidase) to enzymatically hydrolyze adenosine monophosphate (AMP) into adenosine, which then binds to host cell adenosine receptors to trigger the AKT and ERK survival and migration pathways. Furthermore, hDPSC-exosomes carry the lncRNA-Ankrd26, which functions as a “molecular sponge” for miR-150, thereby preventing the degradation of TLR4 transcripts and facilitating the recruitment of mesenchymal stem cells to the injury site.

In contrast, Stem cells from Human Exfoliated Deciduous teeth (SHEDs) produce vesicles with a “biologically younger” profile that favors rapid immunomodulation and revascularization. SHED aggregate exosomes (SA-Exo) are characterized by high levels of miR-26a, which specifically activates the TGF-β/SMAD2/3 signaling axis to drive both the endothelial differentiation of progenitor cells and the recruitment of host microvasculature. When cells undergo programmed death during transplantation, they release apoptotic vesicles (apoVs), which carry the mitochondrial protein TUFM. This protein induces TFEB-mediated autophagy in recipient endothelial cells, an essential metabolic process that provides the energy and structural remodeling required for rapid vessel sprouting in ischemic root canals. Furthermore, under odontogenic induction, SHED-EVs preferentially activate the AMPK/mTOR pathway, a master metabolic regulator that shifts recipient hDPSCs toward an odontogenic phenotype.

Stem cells from the Apical Papilla (SCAPs) emerge as the specialized source for managing the delicate apical microenvironment and ensuring root completion. SCAP-derived exosomes are unique in their ability to promote Tet2-mediated demethylation of the Foxp3 locus, an epigenetic modification that converts CD4 + T cells into regulatory T cells (Tregs), thereby stabilizing the local immune environment and preventing chronic inflammation from stalling the regenerative process. In terms of dentinogenesis, SCAP-Exos exhibit a high degree of specificity for Dentin Sialophosphoprotein (DSPP) induction, which marks late-stage odontoblastic maturation, without prematurely triggering generalized osteogenic pathways.

Supplementary insights from complementary studies further clarify these advantages. Zuo et al. (2025) revealed that SHED-derived small EVs contain 25.9-fold higher levels of miR-200c-3p than adult hDPSC-exosomes [38]. This specific miRNA targets PTEN (a tumor suppressor and inhibitor of growth signaling), leading to the robust activation of the PI3K/Akt pathway to polarize macrophages toward a pro-healing M2 phenotype. Environmental stressors also refine these molecular targets: hypoxic preconditioning (1–2% $O_2$) forces SHED-exosomes to enrich their cargo with let-7f-5p and miR-210-3p, which accelerate vascular tube formation via the let-7f-5p/AGO1/VEGF axis. Simultaneously, hypoxic SCAP-exosomes activate the Notch/JAG1/VEGF cascade, where exosomal JAG1 acts as a ligand to trigger pro-angiogenic Notch signaling in host endothelial cells. These findings support a strategic therapeutic hierarchy: SHED-EVs are the ideal candidates for initial inflammatory suppression and rapid revascularization due to their PI3K/Akt and TGF-β potency, whereas hDPSC/SCAP-EVs are the preferred tools for driving final structural maturation and the functional integration of the pulp-dentin complex through circRNA and enzymatic adenosine signaling.

### Risk of bias, consistency and methodological gaps (factual description)

**Fig. 2:**
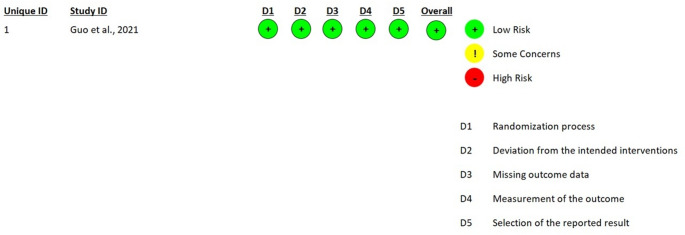
Risk of bias assessment of preclinical studies using the RoB-2 tool

The comprehensive risk-of-bias evaluation revealed a body of evidence that is generally robust but characterized by specific reporting gaps. The RoB 2 assessment for the clinical pilot by Guo et al. (2021) indicates a Low Risk of Bias, supported by clear randomization codes and an exhaustive 24-month follow-up of all 15 patients without missing data [20] (Fig. 2).

**Fig. 3:**
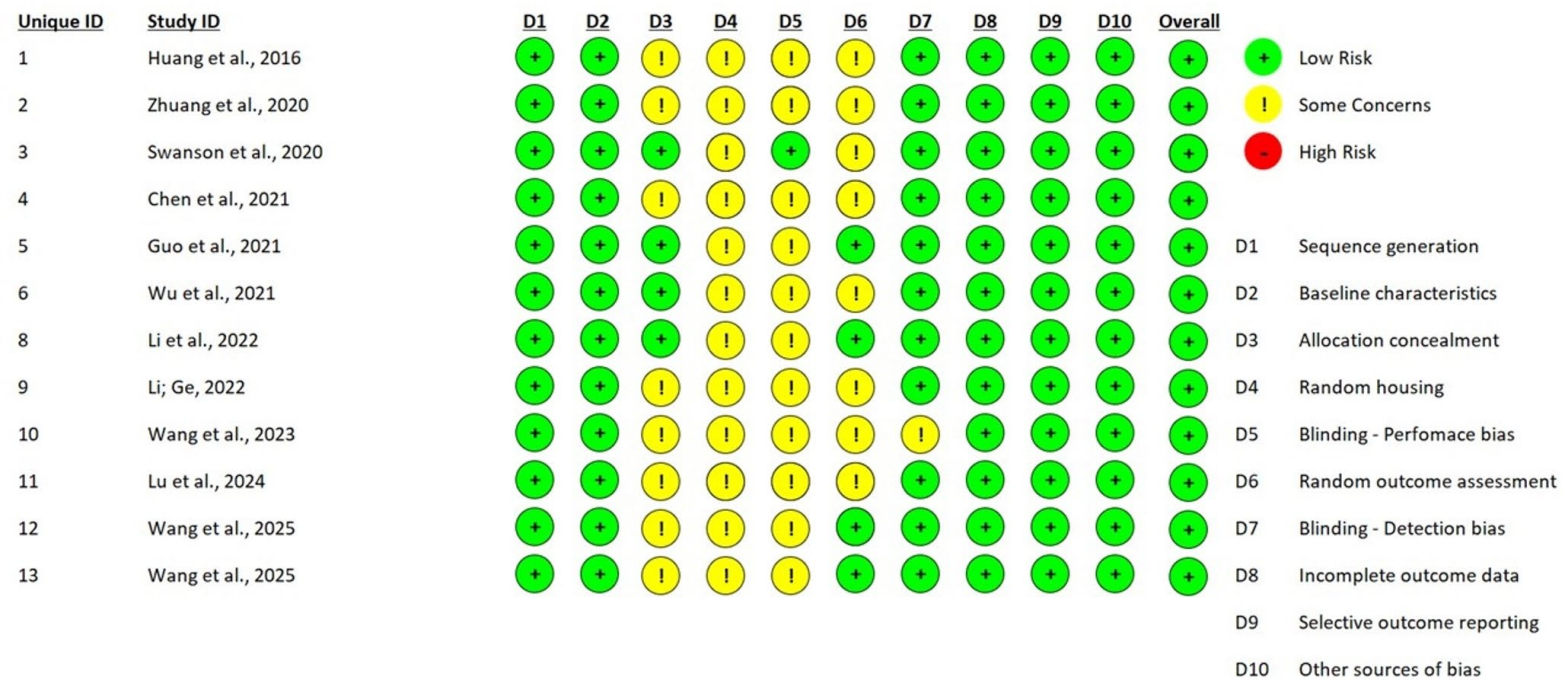
Risk of bias assessment of preclinical studies using the SYRCLE tool

In the SYRCLE evaluation of animal studies, most works demonstrated high quality in outcome measurement (D7) and selective reporting (D9), though many were classified as Unclear Risk regarding random housing (D4) and blinding of personnel (D5), which is a frequent omission in regenerative dentistry literature (Fig. 3).

**Fig. 4:**
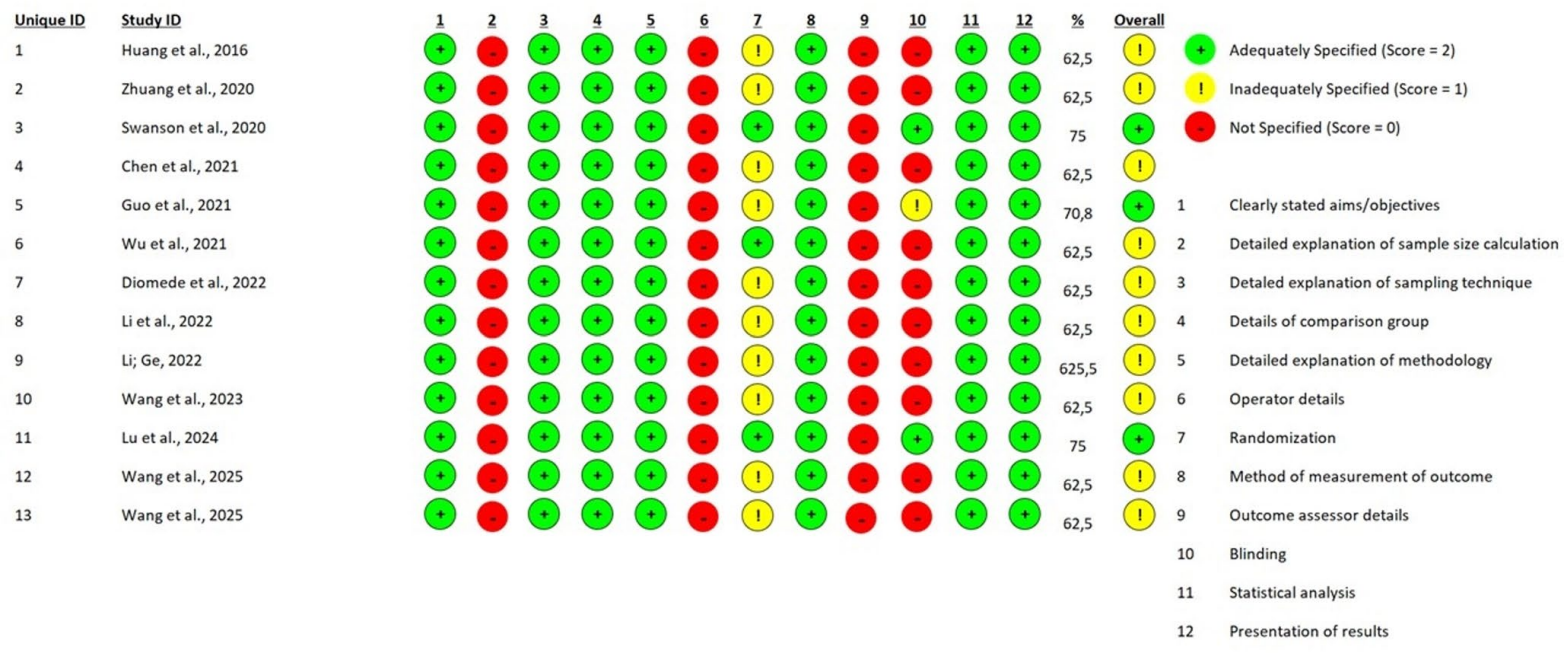
Risk of bias assessment of preclinical studies using the QUIN tool

For the laboratory components, the QUIN tool categorized the studies primarily into Low to Medium Risk (Fig. 4); while methodological detail (Item 5) and statistical analysis (Item 11) were consistently high, nearly all studies failed to provide a formal sample size calculation (Item 2) or details on operator calibration (Item 6). Studies like Swanson et al. (2020) and Lu et al. (2024) achieved higher QUIN scores (75%) by providing superior detail on polymer synthesis, randomization of experimental units, and independent technical replications [26, 31].

A critical limitation identified during this synthesis concerns the definitive causal attribution of the observed regenerative effects. Although studies that compare extracellular vesicles (EVs) against EV-depleted conditioned medium (CM) - such as the benchmark study by Merckx et al. (2020) [32] - provide essential evidence of specificity, the majority of the included works did not incorporate such robust functional controls. Only a minority utilized specific inhibitors of vesicle biogenesis, such as GW4869, or performed “rescue” experiments to confirm that the therapeutic outcome was exclusively particle-mediated. For instance, Wu et al. (2021) and Li & Ge (2022) utilized GW4869 to demonstrate that blocking exosome secretion significantly diminished migration and differentiation, yet the potential contribution of other non-vesicular secretome components (e.g., soluble growth factors) remains under-explored in the broader body of evidence [23, 24]. Consequently, the extent to which the reported pulp-dentin regeneration can be attributed solely to the EV/exosome fraction versus the synergy of the total secretome remains partially uncertain. This limitation reduces the strength of causal inference and highlights the need for future research to adopt parallel comparisons between CM vs. EV vs. EV-depleted CM and rigorous rescue assays to refine cell-free endodontic strategies.

### Critical synthesis of heterogeneity and evidence stability

Qualitative analysis and risk-of-bias assessment revealed marked methodological heterogeneity which, although inherent to the translational and innovative nature of cell-free therapies, imposes major challenges to the stability of conclusions and precludes quantitative synthesis (meta-analysis). This variability can be organized into four critical domains:

#### Heterogeneity of sources and cellular state

Substantial variation was observed in the origin of extracellular vesicles (EVs), encompassing hDPSCs, SHEDs and SCAPs. Distinct biological potencies were identified: EVs from SHEDs exhibited superior immunomodulatory and early revascularization effects via the miR-26a/TGF-β axis, whereas hDPSC- and SCAP-derived EVs were more effective in structural maturation and specific induction of DSPP for tubular dentin formation. Moreover, vesicular “cargoome” bioactivity was markedly altered by preconditioning strategies—such as odontogenic induction, inflammatory priming with LPS, or induction of apoptosis to generate apoVs—complicating the establishment of a universal potency protocol.

#### Variability in isolation protocols and dosing metrics

Although differential ultracentrifugation predominated (12/13 studies), the use of a single commercial precipitation kit in one study introduced potential purity biases and protein co-precipitation that may obscure the true biological effects. More critically, dosing metrics were inconsistently reported—expressed variably as total protein concentration (µg/mL), absolute particle counts by NTA, or yield per million cells—preventing reliable determination of a therapeutic dose–response for clinical translation.

#### Delivery vehicle architecture (Scaffolds)

Progression from “naked” exosome application in collagen matrices to sustained-release platforms (e.g., GelMA hydrogels, chitin-reinforced constructs, PLGA-PEG-PLGA microspheres) substantially alters EV pharmacokinetics. Studies employing bioactive carriers with biomimetic stiffness (≈ 1.06 kPa) reported more organized and consistent repair outcomes than those using free administration, indicating the delivery vehicle is a crucial confounding variable for regenerative endpoints.

#### Fragility of causal attribution and reporting gaps

A key methodological gap is the paucity of robust functional controls: most studies failed to compare isolated EVs against vesicle-depleted conditioned medium or to employ biogenesis inhibitors (e.g., GW4869) in in vivo models. Consequently, exclusive attribution of regenerative effects to the vesicular fraction—rather than to synergy with the soluble secretome—remains partially unresolved. Additionally, widespread absence of sample size calculations and details on operator calibration in in vitro experiments (as flagged by QUIN) downgrades the certainty of evidence from “moderate” to “low.”

While the biological potential of DSC-Exos for pulp–dentin regeneration is evident, variability in characterization protocols (per MISEV guidelines) and lack of dose harmonization warrant caution. Clinical translation therefore depends on standardization of these parameters to ensure reproducible and clinically safe effects.

### Integrated summary by evidence type

The body of evidence for exosome-mediated pulp-dentin regeneration is strategically organized across three experimental hierarchies: in vitro mechanistic validation, in vivo structural reconstruction, and preliminary clinical verification. In vitro research (*n* = 13), encompassing all included primary studies, consistently reports the rapid internalization of extracellular vesicles (EVs) by diverse target cells, including human Dental Pulp Stem Cells (hDPSCs), Bone Marrow Mesenchymal Stem Cells (BMMSCs), and Human Umbilical Vein Endothelial Cells (HUVECs). This uptake triggers immediate biological shifts, characterized by dose-dependent increases in proliferation, migration, and the upregulation of odontogenic/angiogenic markers such as DSPP, DMP-1, and CD31. Studies such as Huang et al. (2016) and Wang et al. (2025) utilize high-resolution techniques, including RT-qPCR, Alizarin Red S mineralization, and Transwell assays, to confirm that exosomes isolated under odontogenic induction are significantly more potent than those from basal states, specifically by activating pathways like p38 MAPK and the circ_0003057/EIF4A3/ANKH axis.

In vivo evidence (*n* = 12) provides the essential structural validation for these cellular effects, utilizing animal models that range from subcutaneous transplantation in nude mice to orthotopic canine and minipig models. Swanson et al. (2020) and Wang et al. (2023) demonstrated that when delivered via controlled-release vehicles like PLGA-PEG microspheres or chitin-reinforced hydrogels, EVs facilitate the formation of organized, vascularized pulp-like tissue and the deposition of tubular dentin bridges [28, 31]. Furthermore, Wu et al. (2021) used exosome inhibitors (GW4869) to prove that the observed revascularization was directly particle-mediated through miR-26a signaling [23]. At the clinical summit, the pilot study (*n* = 1) by Guo et al. (2021) successfully revitalized avulsed teeth in 15 pediatric patients using SHED aggregates [20]. This clinical benchmark demonstrated not only the restoration of thermal and electrical pulp sensitivity but also established long-term systemic safety, as blood profiles and immune counts remained stable over a 24-month follow-up.

### Conclusions from the results

The synthesis of these findings leads to several critical factual interpretations regarding the future of endodontic therapy (Supplementary Fig. 2). First, the cellular source of EVs determines their primary therapeutic switch: SHED-derived vesicles are the optimal choice for rapid immunomodulation and revascularization through the TGF-β/SMAD2/3 pathway and autophagy activation via TUFM protein. In contrast, hDPSC and SCAP-derived vesicles serve as superior drivers for structural maturation, selectively inducing DSPP expression and mineralized matrix organization. Second, the microenvironmental preconditioning (e.g., odontogenic induction or LPS exposure) significantly alters the exosomal “cargome,” turning basal vesicles into high-potency “instructive units” that can redirect host progenitor cells toward a regenerative rather than a reparative (scarring) phenotype.

The immediate implications for clinical practice are profound. These results support a transition toward cell-free, “off-the-shelf” regenerative endodontics, which bypasses the biological risks of tumorigenesis, immune rejection, and the logistical complexities inherent in live cell transplantation. To achieve complete restoration of the pulp-dentin complex, future protocols must adopt multifunctional scaffolds (such as GelMA or decellularized matrices) that mimic the native extracellular matrix stiffness (approx. 1.06 kPa) to support the bioactivity of the exosomal cargo. This strategy ensures the simultaneous recruitment of host endothelial cells and the odontogenic differentiation of resident stem cells, fulfilling the requirements for a functional, vital tooth organ.

Studies excluded from the primary synthesis provide vital refinements to the proposed regenerative models. Excluded works like Merckx et al. (2020) and Zhang et al. (2020) emphasize that while EVs provide superior angiogenic content, they must be contextually delivered with odontogenic cues to ensure the formation of pulp-dentin tissue rather than non-specific connective tissue [32, 34]. The research of Zuo et al. (2025) and Yu et al. (2022) clarifies the immunomodulatory superiority of SHEDs, revealing that their small EVs contain 25.9-fold higher miR-200c-3p than adult sources, which is essential for polarizing macrophages toward a pro-healing M2 phenotype and increasing regulatory T cell (Treg) counts [38, 39]. Furthermore, studies utilizing animal sources or non-pulp MSCs, such as Abdelgawad et al., 2022 and Shi et al. (2023), provide biochemical evidence that combining photobiomodulation (PBM) with EVs can synergistically enhance the mineral levels (calcium and phosphorus) and gene expression of OCN and TGF-β in the blood, suggesting a potent adjuvant for clinical application [40, 42]. These excluded insights collectively suggest that the next phase of research should focus on precision bioengineering, using stressors like hypoxia (1–2% O2) to tailor exosomal cargo (e.g., let-7f-5p, miR-210-3p) for specifically managing ischemic environments in necrotic root canals.

## Discussion

The systematic synthesis of the 13 included studies confirms that dental stem cell-derived extracellular vesicles (DSC-EVs) possess significant biological potential to orchestrate the complex signaling required for pulp-dentin regeneration. The evidence demonstrates a clear transition from in vitro mechanistic validation to in vivo structural reconstruction, culminating in the clinical evidence provided by Guo et al. (2021), who achieved functional revitalization of avulsed permanent teeth in pediatric patients [20]. Mechanistically, the regenerative effect is driven by the delivery of specific non-coding RNAs, such as miR-26a, which activates the TGF-β/SMAD2/3 pathway to promote angiogenesis, and circ_0003057, which facilitates odontogenic differentiation via the EIF4A3/ANKH axis. However, the transition from biological potential to clinical certainty requires addressing several critical methodological and translational gaps.

A primary concern involves the specificity of causal attribution. While the included studies report robust regenerative outcomes, a significant limitation is that most did not incorporate stringent functional controls, such as EV-depleted conditioned medium (CM), exosome biogenesis inhibitors like GW4869, or rigorous “rescue” experiments. Although benchmark research, such as that by Merckx et al. (2020), provides evidence that the vesicular fraction is the primary driver of targeted angiogenesis, the broader body of evidence often fails to distinguish the effects of EVs from the synergy of the total secretome [32]. Only a few studies, such as Wu et al. (2021) and Li & Ge (2022), utilized GW4869 to confirm that blocking secretion significantly diminished cellular migration and differentiation [23, 24]. This limitation reduces the strength of causal inference and necessitates that future research adopt parallel comparisons (CM vs. EV vs. EV-depleted CM) and validated rescue assays to definitively isolate particle-mediated effects.

Furthermore, the methodological quality assessed via RoB 2, SYRCLE, and QUIN tools highlights consistent reporting deficiencies. While the clinical data from Guo et al. (2021) achieved a Low Risk of Bias, the preclinical animal studies frequently omitted details regarding random housing (D4) and blinding of personnel (D5) [20]. Similarly, the QUIN tool revealed that nearly all in vitro dental studies lack formal sample size calculations (Item 2) and details on operator calibration (Item 6). These reporting gaps, combined with the extreme heterogeneity in EV isolation methods (ranging from ultracentrifugation to commercial precipitation kits), dosage metrics, and the diverse mechanical properties of delivery vehicles (e.g., GelMA, chitin hydrogels, PLGA microspheres), prevent the performance of a meta-analysis and warrant translational caution.

To guide future translational strategies, this review establishes a strategic therapeutic hierarchy based on clinical scenarios. For immature permanent teeth or cases requiring rapid revascularization and immunomodulation (e.g., pulp necrosis), SHED-derived vesicles are the preferred biological engine due to their superior PI3K/Akt signaling and ability to polarize macrophages toward a pro-healing M2 phenotype, as supported by Zuo et al. (2025) and Yu et al. (2022) [38, 39]. Conversely, for mature teeth or pulp capping scenarios where the goal is the formation of a localized dentin bridge, hDPSC and SCAP-derived vesicles serve as superior drivers of structural maturation and DSPP-mediated mineralization. The choice of vehicle is equally critical; biomimetic systems like GelMA or chitin-based hydrogels with a native-like stiffness of 1.06 kPa are essential to protect EVs from proteolytic degradation and ensure sustained bioactivity.

Finally, significant translational bottlenecks remain regarding the development of EVs as standardized therapeutic products. Future research must prioritize Good Manufacturing Practice (GMP) production, establishing standardized quality control protocols to manage batch-to-batch variability and ensuring dosage consistency. Aligning experimental protocols with MISEV guidelines and registering data on platforms like EV-TRACK will be essential for regulatory approval. Addressing these regulatory and technical constraints is a prerequisite for advancing toward long-term, controlled clinical trials that can move cell-free endodontics from experimental potential to standard practice.

## Conclusion

In conclusion, the integration of 13 primary studies and 13 complementary works establishes that dental stem cell-derived exosomes represent a highly promising biomimetic tool for pulp-dentin regeneration. The evidence consistently demonstrates their ability to promote odontogenesis, angiogenesis, and neurovascular integration through defined molecular axes like miR-26a/TGF-β and circ_0003057/ANKH. However, the certainty of the evidence remains moderate-to-low due to substantial methodological heterogeneity and the lack of robust functional controls to confirm absolute causal attribution to the vesicular fraction.

The clinical translation of this technology depends on a staged research pathway focused on the precision bioengineering of EV cargo and the standardization of GMP-compliant production. Immediate clinical implications support a transition toward cell-free, “off-the-shelf” protocols using SHED-EVs for revascularization and hDPSC-EVs for structural repair. Moving forward, the field must prioritize experimental harmonization, adherence to MISEV standards, and the validation of adequately powered clinical trials to ensure the safe and effective restoration of the functional pulp-dentin complex.

## Supplementary Information

Below is the link to the electronic supplementary material.

Supplementary Material 1

Supplementary Material 2

Supplementary Material 3

Supplementary Material 4

Supplementary Material 5

Supplementary Material 6

Supplementary Material 7

## Data Availability

The data that support the findings of this study are available from the corresponding author, upon reasonable request.
