# Correction: Efficacy of dental stem cell–derived exosomes for pulp regeneration: a systematic review of clinical, animal, and in vitro studies

**DOI:** 10.1007/s11033-026-11720-2

**Published:** 2026-03-31

**Authors:** Julia Godoi-Lopes, Victor Hugo Alves Ribeiro-Silva, Larissa Gregório Candido do Prado, Igor Bassi Ferreira Petean, Lais Valencise Magri, Fabiane Carneiro Lopes-Olhê, Carla Renata Sipert, Jardel Francisco Mazzi-Chaves

**Affiliations:** 1 Department of Restorative Dentistry, School of Dentistry of Ribeirão Preto, University of São Paulo, São Paulo, Brazil; 2 Department of Restorative Dentistry, School of Dentistry, University of São Paulo, São Paulo, Brazil; 3 Department of Endodontics Ribeirão Preto School of Dentistry, University of São Paulo (USP), Av. do Café, s/n,Ribeirão Preto, São Paulo, 14020-904 Brazil

**Correction to: Molecular Biology Reports (2026) 53:426**

10.1007/s11033-026-11547-x

In this article, Fig. 1 appeared incorrectly and has now been corrected in the original publication. For completeness and transparency, the correct and old incorrect versions are displayed below. 

Incorrect Fig. 1:

**Fig. 1:**
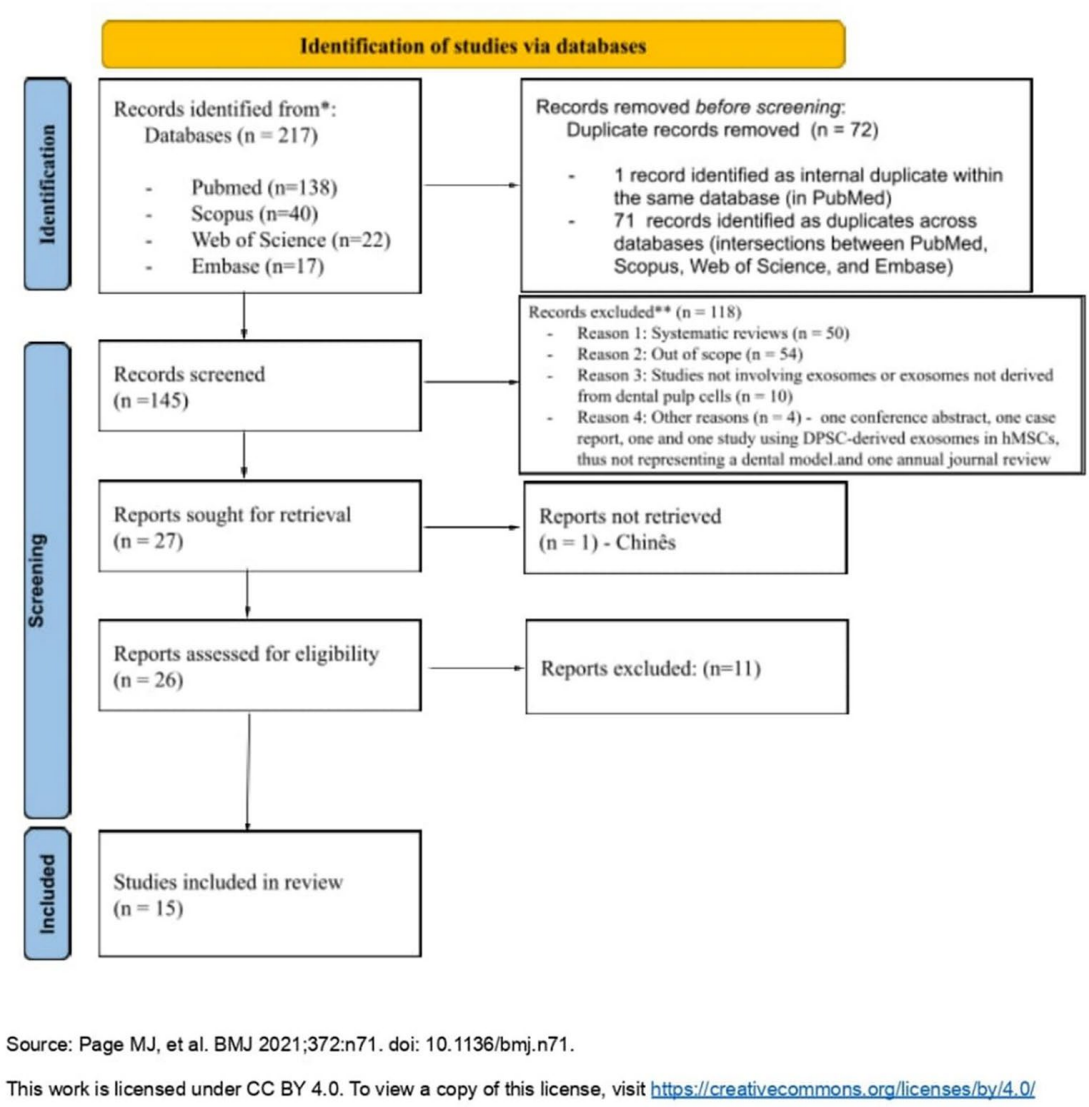
PRISMA flow diagram of study selection

Correct Fig. 1.

**Fig. 1:**
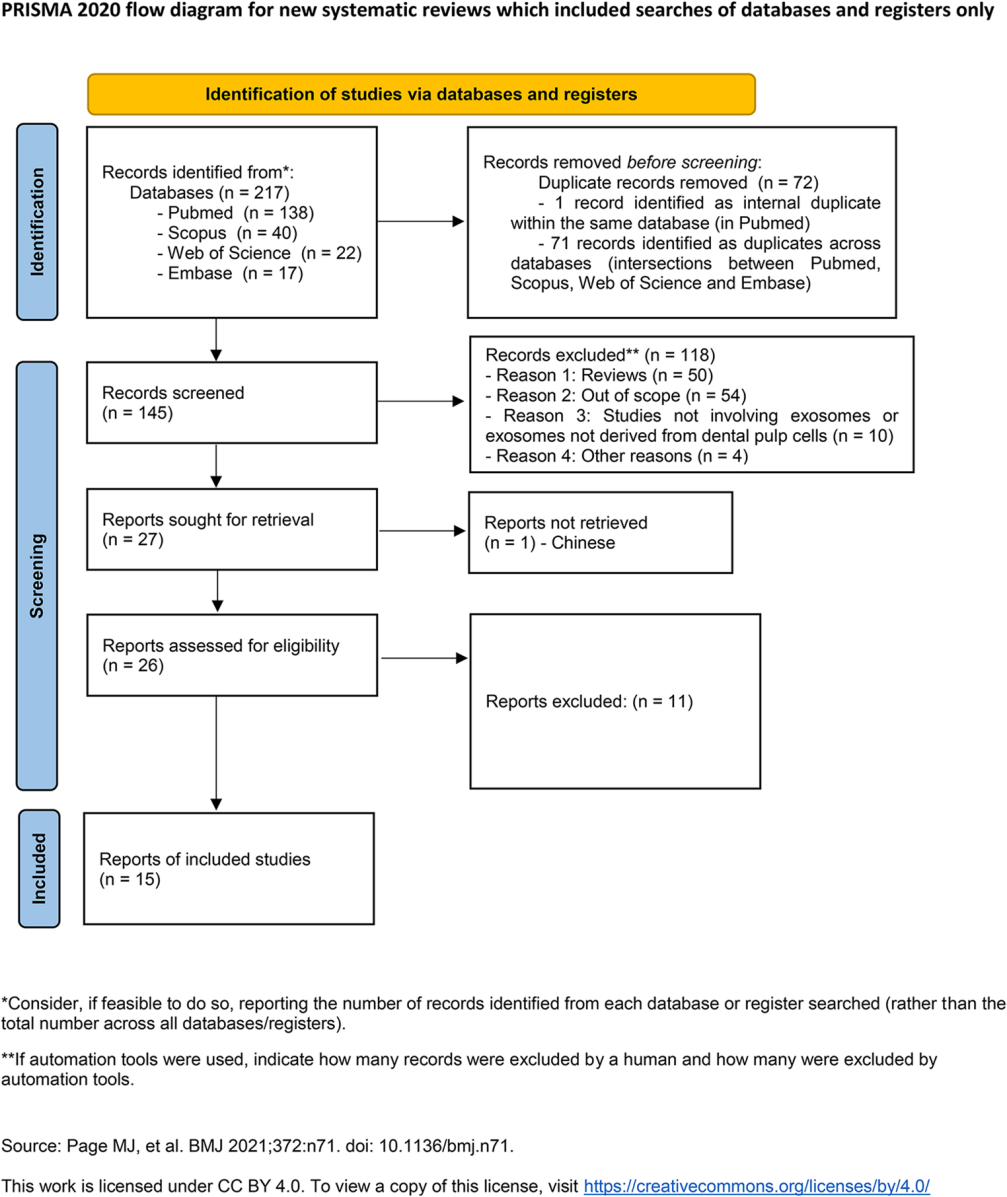
PRISMA flow diagram of study selection

The original article has been corrected.

