# Supplementary material for: Efficacy of dental stem cell–derived exosomes for pulp regeneration: a systematic review of clinical, animal, and in vitro studies
Source: Mol Biol Rep. 2026 Feb 24;53(1):426. doi: 10.1007/s11033-026-11547-x (PMC12932340; doi:10.1007/s11033-026-11547-x)
Supplement: Supplementary file 1 — Supplementary Material 1 [file 11033_2026_11547_MOESM1_ESM.docx]

**Supplementary Table S1**. Search strategies used for database queries, including number of hits and date of search.

| **Database** | **Search strategy** | **Records retrieved** | **Date of last search** |
| --- | --- | --- | --- |
| **PubMed**  ***(Via NCBI)*** | ((((((Exosomes) OR (exosome)) OR (extracellular vesicles)) OR (EVs)) AND ((((((Stem Cells) OR (dental stem cells)) OR (DPSCs)) OR (SHED)) OR (SCAP)) OR (PDLSCs))) AND ((((Pulp Regeneration) OR (pulp regeneration)) OR (regenerative endodontics)) OR (pulp tissue engineering))) AND (((tooth) OR (teeth)) OR (dental pulp)) | 138 | 29/07/2025 |
| **Scopus**  ***(Elsevier)*** | (TITLE-ABS-KEY ( exosomes  OR  exosome  OR  endossome  OR  "extracellular vesicles"  OR  ev )  AND  TITLE-ABS-KEY ( "stem cell"  OR  "dental stem cells"  OR  dpscs  OR  shed  OR  scap  OR  pdlsc )  AND  TITLE-ABS-KEY ( "pulp regeneration"  OR  "regenerative endodontics"  OR  "pulp tissue engineering" ) ) | 40 | 29/07/2025 |
| **Web of Science *(Clarivate)*** | TS=(exosomes OR "extracellular vesicles") AND TS=("dental stem cells" OR "dental pulp stem cells" OR DPSCs OR SHED OR SCAP OR PDLSCs OR "stem cells from human exfoliated deciduous teeth") AND TS=("pulp regeneration" OR "regenerative endodontics" OR "pulp tissue engineering" OR "dental pulp healing") | 22 | 29/07/2025 |
| **Embase**  ***(Elsevier/***  ***Embase.com)*** | ('exosome'/exp OR exosomes OR 'extracellular vesicles' OR EVs) AND ('dental stem cell'/exp OR 'dental stem cells' OR DPSCs OR SHED OR SCAP OR PDLSCs) AND ('pulp regeneration'/exp OR 'pulp regeneration' OR 'regenerative endodontics' OR 'pulp tissue engineering') | 17 | 29/07/2025 |

Note: Searches run on 29 July 2025. No language or date filters were applied. Access provided via CAPES/CAFe consortium (CNPq, Brazil)
