# Supplementary material for: Efficacy of dental stem cell–derived exosomes for pulp regeneration: a systematic review of clinical, animal, and in vitro studies
Source: Mol Biol Rep. 2026 Feb 24;53(1):426. doi: 10.1007/s11033-026-11547-x (PMC12932340; doi:10.1007/s11033-026-11547-x)
Supplement: Supplementary file 2 — Supplementary Material 2 [file 11033_2026_11547_MOESM2_ESM.docx]

**Supplementary Table S4.** Evidence from studies on SHED-derived exosomes.

| **Author, Year** | **Model / Design** | **Intervention / EV Source** | **Main Outcomes** | **Mechanistic Insights** |
| --- | --- | --- | --- | --- |
| **Wu et al., 2021** | *In vitro (2D assays) + In vivo (ectopic subcutaneous human root fragments in nude mice)* | SHED aggregate exosomes (SA-Exo) | Enhanced dentin-pulp complex regeneration with increased CD31+ vessel density | SA-Exos are enriched with miR-26a, which activates the TGF-β/SMAD2/3 signaling pathway for angiogenesis |
| **Guo et al., 2021** | *In vitro (2D aggregates) + In vivo (minipig avulsion model) + Human clinical pilot* | hDPSC aggregates (SHED-derived) + Decellularized Tooth Matrix (DTM) | Clinical restoration of thermal/electrical sensitivity and continued root development in children. | DTM reconstructs an odontogenic niche that induces exosome release, activating developmental gene axes (DMP4/DLX1) |
| **Li et al., 2022** | *In vitro (2D assays) + In vivo (orthotopic beagle dog pulp ablation and revascularization)* | SHED-derived apoptotic vesicles (apoVs) | Significant revascularization and vital pulp-like tissue formation in beagles. | apoVs transport mitochondrial factor TUFM, activating the TFEB-autophagy pathway in recipient endothelial cells |
| **Lu et al., 2024** | *In vitro (2D assays) + In vivo (ectopic subcutaneous human root fragments in nude mice)* | Pre-differentiated SHED-EVs (OM-EV) in GelMA hydrogel | Promoted dense mineralized tissue formation and high expression of DSPP and DMP-1 | Upregulation of AMPK and downregulation of mTOR signaling identified via microRNA sequencing. |
| **Liu et al., 2023 (complementary)** | *In vitro (2D HUVECs) + In vivo (murine matrigel plug assay)* | Hypoxia-conditioned SHED-Exos (2% O2) | Significantly enhanced micro-vessel formation and higher VEGF expression in vivo | Transfer of let-7f-5p and miR-210-3p to regulate angiogenesis via AGO1/VEGF and ephrinA3 axes |
| **Zuo et al., 2025 (complementary)** | *In vitro (cloned murine and mouse peritoneal macrophages)* | SHED-sEVs (Young donors vs. Adult) | Superior induction of pro-healing M2 macrophage polarization compared to adult DPSCs | SHED-sEVs contain 25.9-fold higher miR-200c-3p, targeting PTEN to activate the PI3K/Akt pathway |
