# Supplementary material for: Efficacy of dental stem cell–derived exosomes for pulp regeneration: a systematic review of clinical, animal, and in vitro studies
Source: Mol Biol Rep. 2026 Feb 24;53(1):426. doi: 10.1007/s11033-026-11547-x (PMC12932340; doi:10.1007/s11033-026-11547-x)
Supplement: Supplementary file 3 — Supplementary Material 3 [file 11033_2026_11547_MOESM3_ESM.docx]

**Supplementary Table S5.** Evidence from studies on SCAP-derived exosomes.

| **Author, Year** | **Model / Design** | **EV Source / Intervention** | **Main Outcomes** | **Mechanistic Insights** |
| --- | --- | --- | --- | --- |
| **Zhuang et al., 2020** | *In vitro (2D assays with rat BMMSCs) + In vivo (ectopic subcutaneous human root fragments in nude mice)* | hSCAP-Exos (Human Stem Cells from Apical Papilla) isolated via ultracentrifugation | Promoted specific dentinogenesis and vascularity; formation of a continuous dentine layer with polarized odontoblast-like cells | Specifically upregulated DSPP (Dentin Sialophosphoprotein) expression in recipient cells without affecting general osteogenic markers like ALP |
| **Yu et al., 2022 (complementary)** | *In vitro (CD4+CD25- T cell assays) + In vivo (orthotopic rat molar model of experimentally induced pulpitis)* | hSCAP-Exos isolated via ultracentrifugation | Effectively alleviated pulp inflammation and promoted the accumulation of Foxp3+ regulatory T cells (Tregs) in situ | Promoted Tet2-mediated Foxp3 demethylation to stabilize Treg conversion and increased IL-10 production |
| **Liu et al., 2022 (complementary)** | *In vitro (2D HUVEC assays: proliferation, migration, and tube formation)* | Hypoxia-conditioned hSCAP-Exos (1% O2) | Significantly accelerated endothelial proliferation and formation of capillary-like network structures | Activated the HIF-1α/Notch1/JAG1/VEGF signaling cascade via exosomal delivery of the JAG1 protein |
