# Supplementary material for: Efficacy of dental stem cell–derived exosomes for pulp regeneration: a systematic review of clinical, animal, and in vitro studies
Source: Mol Biol Rep. 2026 Feb 24;53(1):426. doi: 10.1007/s11033-026-11547-x (PMC12932340; doi:10.1007/s11033-026-11547-x)
Supplement: Supplementary file 4 — Supplementary Material 4 [file 11033_2026_11547_MOESM4_ESM.docx]

**Supplementary Table S2.** Excluded but contextually relevant studies and their methodological/molecular contributions.

| Author, Year | Model / Cell Source | Reason for Exclusion (per protocol) | Key Methodological / Mechanistic Contributions |
| --- | --- | --- | --- |
| Merckx et al., 2020 | Human DPSC and BM-MSC | Primary focus on comparing angiogenic content and functions rather than complete pulp-dentin tissue formation | Identified that while all fractions induce endothelial motility, EV-depleted conditioned medium (soluble factors) exhibits stronger angiogenic potential in ovo |
| Ivica et al., 2020 | Human DPC (Total Population) | Uses a heterogeneous population of dental pulp cells (DPC) rather than a pure stem cell lineage. | Demonstrated that pulp-derived exosomes in a fibrin sealant attract MSCs and enhance their proliferation for "cell homing" endodontics |
| Zhang et al., 2020 | Human DPSC | Specific focus on neovascularization (angiogenesis) instead of full pulp-dentin structural reconstruction. | Developed an injectable EV-fibrin gel composite that supports rapid vascular-like formation (7 days) and deposition of collagen IV. |
| Liu et al., 2023 | Human SHED | Focus restricted to enhanced angiogenesis/micro-vessel formation. | Confirmed that hypoxia preconditioning (2%) enriches SHED-Exos with let-7f-5p and miR-210-3p to regulate angiogenesis via AGO1/VEGF axes. |
| Yu et al., 2022 | Human SCAPs | Focuses primarily on immunomodulation and alleviating pulpitis via T-cells/Tregs. | Demonstrated that SCAP-Exos promote Tet2-mediated Foxp3 demethylation to stabilize Tregs and suppress dental pulp inflammation |
| Abdelgawad et al., 2022 | Canine (dog) DPSCs. | Utilizes a non-human (animal) source for exosome isolation. | Reported that combining photobiomodulation (PBM, 980nm) with DPSC exosomes synergistically enhances blood mineral levels (Ca/P) and OCN expression. |
| Chen et al., 2022 | Swine dental pulp tissue (DPT) and DPCs | Utilizes animal tissues and swine cell sources (Miniature Swine Model) | Proved that pulp tissue-derived exosomes (DPT-exos) are superior to cell-derived EVs in recruiting SCAPs for vascularized pulp-like tissue formation. |
| Yan et al., 2022 | Human DPSC and PDLSC | Focuses on the differentiation of Periodontal Ligament Stem Cells (PDLSCs), not pulp-dentin complex | Showed that inflammatory DPSC-EVs deliver miR-758-5p to target LMBR1 and activate BMP signaling for PDLSC differentiation. |
| Yang et al., 2022 | Rat DPC and SCAP | Focuses on treating apical periodontitis (periapical destruction) rather than intracanalar regeneration. | Engineered NFIC-encapsulated EVs using HEK293FT cells to promote SCAP migration and dentinogenesis in vitro and in vivo. |
| Ganesh et al., 2023 | Rabbit DPSC | Utilizes a non-human (animal) source for exosome isolation. | Utilized NGS to identify exosomal miR-199a-3p, 221-3p, and 21-5p as critical regulators of cell homing and angiogenic differentiation |
| Shi et al., 2023 | Human hESC-MSC | The source of EVs is embryonic stem cell-derived MSCs, not of pulpal origin. | Discovered that MSC exosome bioactivity is mediated by exosomal CD73, which activates adenosine receptors to trigger AKT/ERK signaling in pulp cells. |
| Liu et al., 2022 | Human SCAP | Strict focus on accelerating in vitro angiogenesis via Notch/JAG1 | Elucidated that hypoxic SCAP-Exos deliver the JAG1 protein to activate the HIF-1α/Notch1/VEGF signaling cascade in endothelial cells |
| Zuo et al., 2025 | Human SHED and DPSCs | Primary focus on immunomodulatory mechanisms (macrophage polarization) rather than pulp regeneration | Found SHED-sEVs contain 25.9-fold higher miR-200c-3p than adult sources, targeting PTEN/PI3K/Akt to drive pro-healing M2 polarization. |
