# Supplementary figures and images for: Efficacy of dental stem cell–derived exosomes for pulp regeneration: a systematic review of clinical, animal, and in vitro studies
Source: Mol Biol Rep. 2026 Feb 24;53(1):426. doi: 10.1007/s11033-026-11547-x (PMC12932340; doi:10.1007/s11033-026-11547-x)

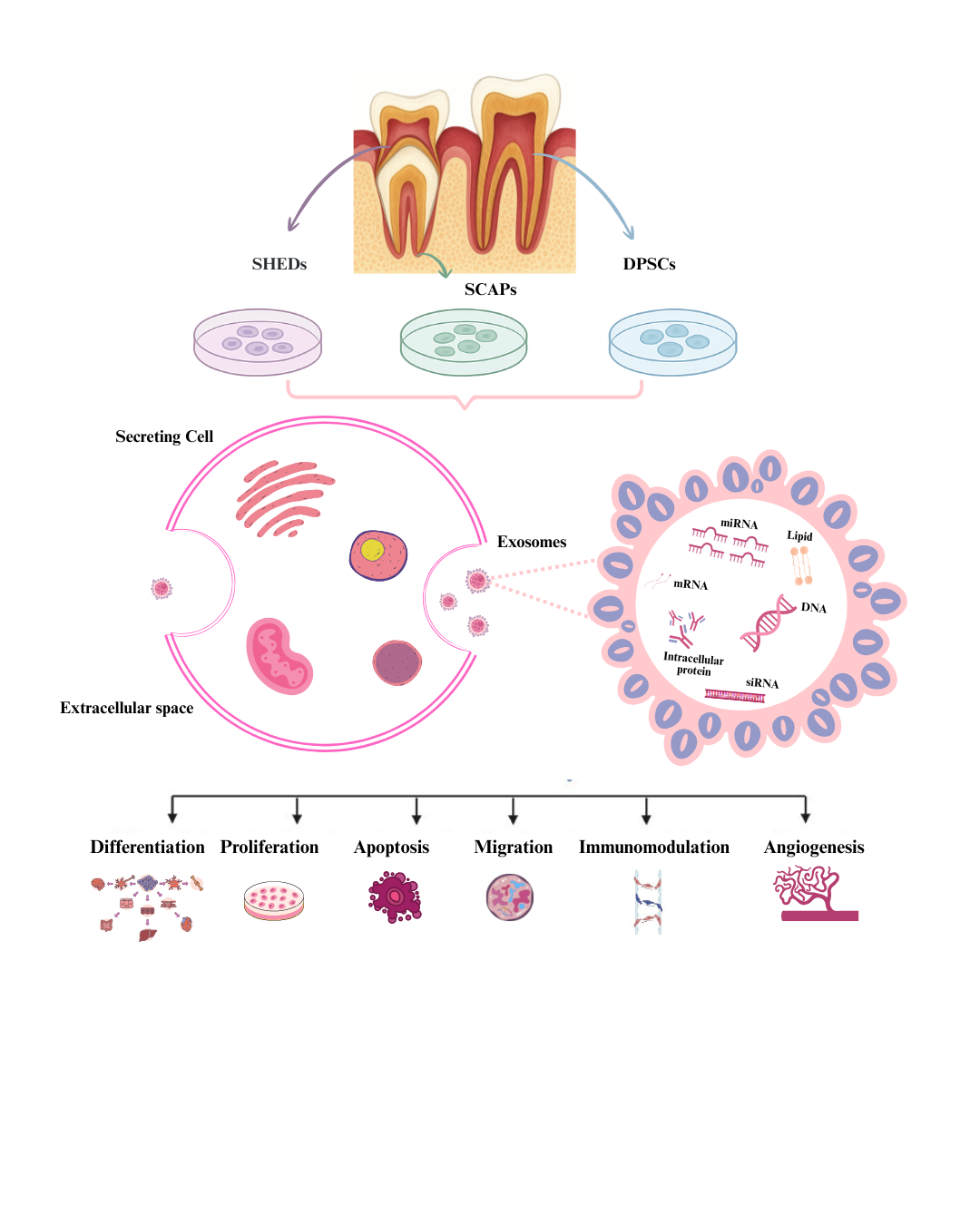

Supplement: Supplementary file 5 — Supplementary Material 5 [file 11033_2026_11547_MOESM5_ESM.png]

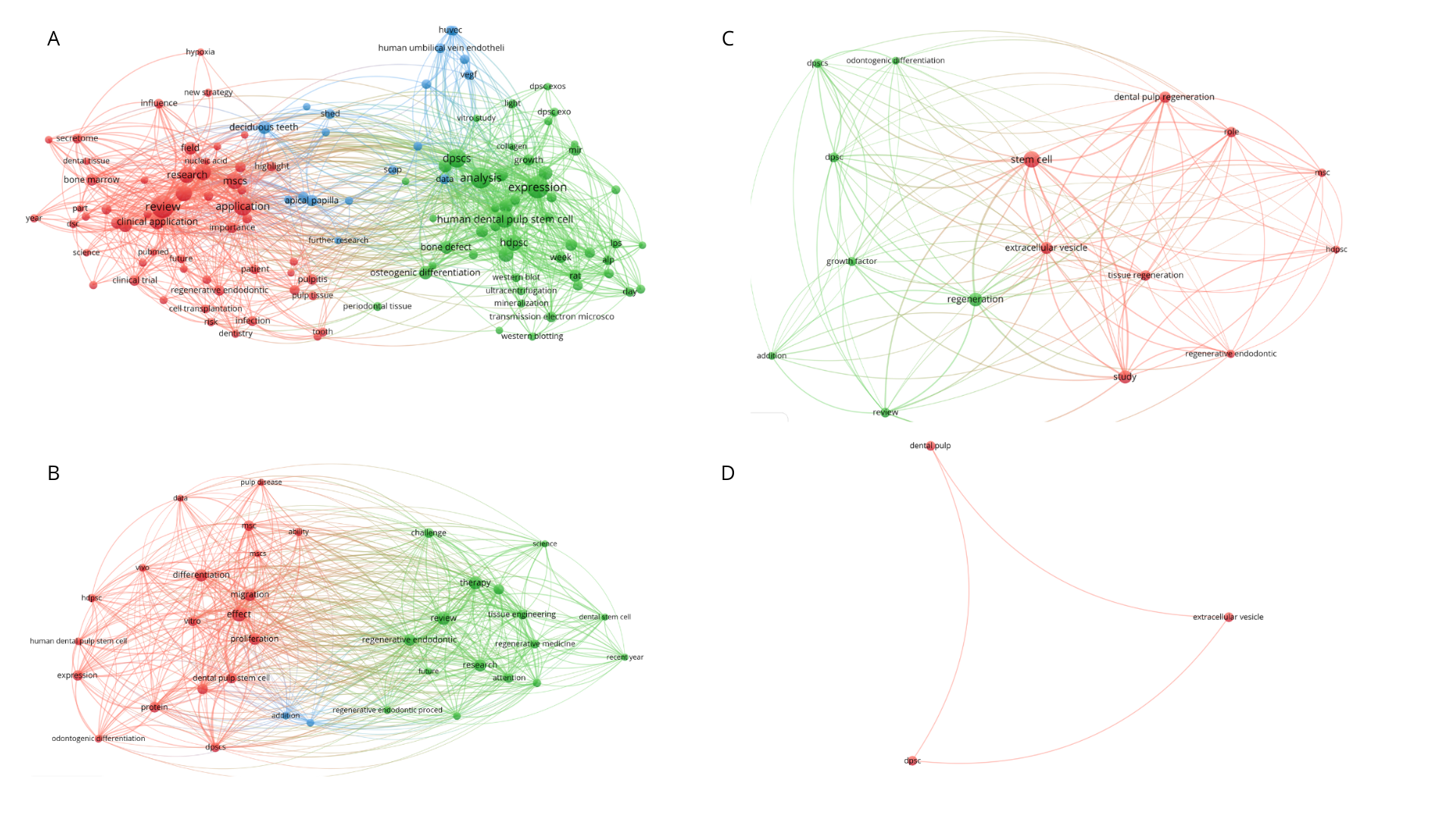

Supplement: Supplementary file 7 — Supplementary Material 7 [file 11033_2026_11547_MOESM7_ESM.png]
