# Supplementary material for: Efficacy of dental stem cell–derived exosomes for pulp regeneration: a systematic review of clinical, animal, and in vitro studies
Source: Mol Biol Rep. 2026 Feb 24;53(1):426. doi: 10.1007/s11033-026-11547-x (PMC12932340; doi:10.1007/s11033-026-11547-x)
Supplement: Supplementary file 6 — Supplementary Material 6 [file 11033_2026_11547_MOESM6_ESM.docx]

**Supplementary Table S3.** Evidence from studies on DPSC-derived exosomes.

| **Author, Year** | **Model / Design** | **EV Source / Intervention** | **Main Outcomes** | **Mechanistic Insights** |
| --- | --- | --- | --- | --- |
| **Huang et al., 2016** | *In vitro (2D/3D culture) + In vivo (ectopic human root slice in nude mice)*. | hDPSC-Exos (growth vs. odontogenic induction) | Upregulated odontogenic genes and mineralized nodules; triggered pulp-like tissue in vivo | Caveolar endocytosis triggers P38 MAPK and upregulates BMP2/BMP9 |
| **Swanson et al., 2020** | *In vitro (2D assays) + In vivo (ectopic nude mice & orthotopic rat pulpotomy)* | hDPSC and MDPC-Exos in PLGA-PEG-PLGA microspheres | Formed organized tubular reparative dentin bridge at exposure sites | Activation of Erk1/2 MAPK signaling and RUNX2 protein expression |
| **Chen et al., 2021** | *In vitro (2D assays) + In vivo (rat pulpless canal model)* | hDPSC-sEVs (LPS preconditioned). | Functional healing with vascularized loose connective tissue; superior outcome compared to non-primed EVs | LPS stimulation facilitates increased exosome yield and induces a pro-healing phenotype via TLR4 activation |
| **Diomede et al., 2022** | *In vitro recellularization* | hDPSC-EVs + 5-Azacytidine + Decellularized Pulp (DDP) | Enhanced recellularization of DDP matrix and expression of dentinogenesis markers (DSPP/DMP1). | Synergy between EVs and 5-Aza promotes DNA demethylation of odontogenic differentiation-associated genes |
| **Li & Ge, 2022** | *In vitro and In vivo (Rat injury model)* | hDPSC-Exos (lncRNA-Ankrd26) | Promoted migration and osteoblastic differentiation of host MSCs for pulp restoration | Operates through the lncRNA-Ankrd26/miR-150/TLR4 regulatory axis |
| **Wang et al., 2023** | *In vitro and In vivo (Nude mice)* | hDPSC-Exos (circ_0003057) | Significant promotion of osteo/odontogenic differentiation and thicker dentin-like tissue formation | circ_0003057 binds to EIF4A3, facilitating its nuclear export and upregulating the parental gene ANKH. |
| **Wang et al., 2025** | In vivo (Human root fragments) | hDPSC-Od-Exos (Early pre-differentiation) | Complete pulp-dentin complex regeneration with neurovascular networks and tubular dentindentin, vessels, nerves). | Recapitulates the developmental microenvironment by enriching exosomes with cues for odontogenesis, angiogenesis, and neurogenesis. |
| **Wang et al., 2025** | *In vitro* (3D osteo/odontogenic differentiation assays) + *In vivo* (subcutaneous implantation of dentin fragments in nude mice). | hDPSC exosomes time-profiled (EXO-7d enriched in circ_0003057); TEM/NTA/WB. | XO-7d → ↑ ALP activity, ↑ ARS mineralization; ↑ RUNX2/OCN/DSPP/DMP1; *in vivo* → increased dentin-like matrix formation. | Exosomal circ_0003057 binds EIF4A3 to stabilize/export ANKH mRNA → ANKH upregulation drives mineralization programs. |
| **Merckx et al., 2020 (complementary)** | *In vitro (2D HUVECs) + In ovo (chicken CAM assay)* | hDPSC vs. BM-MSC vesicles | hDPSCs were less potent in neovascularization compared to bone marrow sources | Found that soluble factors in conditioned medium dominate the angiogenic effect over isolated EVs |
| **Ivica et al. 2020 (complementary)** | *In vitro (3D fibrin gel recellularization)* | Human Total DPC exosomes | Significant attraction of host MSCs and enhanced cell proliferation | Fibrin sealant acts as a synergistic delivery system for exosome-mediated recruitment |
| **Zhang et al. 2020 (complementary)** | *In vitro (3D fibrin gel and 2D assays)* | hDPSC-EVs | Supported rapid neovascularization (<7 days) and collagen (I, III, IV) deposition | EVs stimulate cells to secrete VEGF and carry synergistic cues like FGFb and Leptin |
| **Yan et al. 2022 (complementary)** | *In vitro (PDLSC induction)* | iDPSC-EVs (Inflammatory) | Inflammatory EVs enhanced osteogenic/odontogenic differentiation of neighboring cells | Delivery of miR-758-5p targets LMBR1 to activate the BMP signaling pathway |
| **Ganesh et al. 2023 (complementary)** | *In vitro (Rabbit DPSC migration and differentiation)* | Rabbit DPSC-Exos (Growth vs. Induction) | Upregulation of angiogenic genes (VEGFA, FLT1, PECAM1) in recipient cells | Identification of miR-199a-3p and 21-5p as key exosomal regulators for revascularization |
|  |  |  |  |  |
